# The Lipid Transfer Protein CERT Interacts with the *Chlamydia* Inclusion Protein IncD and Participates to ER-*Chlamydia* Inclusion Membrane Contact Sites

**DOI:** 10.1371/journal.ppat.1002092

**Published:** 2011-06-23

**Authors:** Isabelle Derré, Rachel Swiss, Hervé Agaisse

**Affiliations:** Section of Microbial Pathogenesis, Yale University School of Medicine, New Haven, Connecticut, United States of America; Duke University, United States of America

## Abstract

Bacterial pathogens that reside in membrane bound compartment manipulate the host cell machinery to establish and maintain their intracellular niche. The hijacking of inter-organelle vesicular trafficking through the targeting of small GTPases or SNARE proteins has been well established. Here, we show that intracellular pathogens also establish direct membrane contact sites with organelles and exploit non-vesicular transport machinery. We identified the ER-to-Golgi ceramide transfer protein CERT as a host cell factor specifically recruited to the inclusion, a membrane-bound compartment harboring the obligate intracellular pathogen *Chlamydia trachomatis*. We further showed that CERT recruitment to the inclusion correlated with the recruitment of VAPA/B-positive tubules in close proximity of the inclusion membrane, suggesting that ER-Inclusion membrane contact sites are formed upon *C. trachomatis* infection. Moreover, we identified the *C. trachomatis* effector protein IncD as a specific binding partner for CERT. Finally we showed that depletion of either CERT or the VAP proteins impaired bacterial development. We propose that the presence of IncD, CERT, VAPA/B, and potentially additional host and/or bacterial factors, at points of contact between the ER and the inclusion membrane provides a specialized metabolic and/or signaling microenvironment favorable to bacterial development.

## Author Summary

The obligate intracellular bacterial pathogen *Chlamydia* has developed strategies to invade, survive and replicate within the host genital, ocular and pulmonary epithelial surfaces. *Chlamydia* developmental cycle occurs in a membrane bound vacuole, the inclusion. The *Chlamydia*-dependent remodeling of the inclusion membrane leads to a unique and specialized compartment that allows for the specific interaction with cellular organelles and the acquisition of molecules and nutrients required for bacterial survival and replication. This study provides an example of how the insertion of *C. trachomatis* proteins into the inclusion membrane may allow the bacteria to manipulate the host cells to its own benefit. We showed that the lipid transfer protein CERT localized to *C. trachomatis* inclusion membrane and partly co-localized with the endoplasmic reticulum (ER) protein VAPB. Moreover, VAPB positive tubules made close contact with the inclusion membrane, suggesting the formation of ER-Inclusion membrane contact sites upon *C. trachomatis* infection. Finally, we have shown that CERT interacted with the *C. trachomatis* inclusion protein IncD. We propose that the presence of CERT, VAPB and IncD at ER-Inclusion membrane contact sites allow *C. trachomatis* to exploit the non-vesicular lipid transport machinery of the host cell and generate platforms specialized in metabolism and signaling events favorable to bacterial development.

## Introduction

*Chlamydia* species are obligate intracellular Gram-negative bacterial pathogens that infect genital, ocular and pulmonary epithelial surfaces. *Chlamydia* are characterized by a biphasic developmental cycle that occurs exclusively in the host cell. The bacteria alternate between an infectious, metabolically inactive form called elementary body (EB) that is characterized by a condensed nucleoid, and an intracellular, metabolically active form named reticulate body (RB). Once internalized, *Chlamydia* resides in a membrane bound compartment, named the inclusion. Shortly after uptake, an uncharacterized switch occurs leading to the differentiation of EBs into RBs. The RBs then start to replicate until the inclusion occupies a large part of the cytosol of the host cell. Midway through, the developmental cycle becomes asynchronous and RBs start to differentiate back into EBs. At the end of the cycle, which last two to three days depending on the species, EBs are released from the host cell allowing infection of neighboring cells [1], [2].

To establish and maintain their intracellular niche, *Chlamydia* manipulate the host cellular machinery [3]. Once internalized [4]–[6], *Chlamydia* directs the trafficking of the nascent inclusion to a perinuclear localization *via* a mechanism involving microfilaments, microtubules and the motor protein dynein [7]. The inclusion is encased in a scaffold of host actin and intermediate filaments [8] and infection induces Golgi fragmentation and formation of Golgi ministacks that surround the inclusion [9]. ER tracks containing *Chlamydia* antigens have also been described in the vicinity of the inclusion membrane [10]. The inclusion does not interact with the endocytic pathway [7], [11], however it intercepts exocytic vesicles and lipids from the Golgi [12]. Resident protein and lipid constituents of multivesicular bodies are also delivered to *Chlamydia* inclusion [13], [14]. Some Rab GTPases are recruited to the inclusion membrane [15], and host lipid droplets are targeted to enhance intracellular survival and replication [16], [17].

The secretion of *Chlamydia* effectors proteins is thought to be important for a successful developmental cycle. To date, many *Chlamydia* effector proteins have been identified. Some, but not all, are type III secretion substrates and their function range from entry to establishing and maintaining the replicative vacuole [18], [19]. Some of these effectors are released into the host cell cytosol where they target cellular organelles or signaling pathways, while others act directly at the inclusion membrane. *C. trachomatis* encodes ∼50 putative inclusion membrane proteins, including the Inc proteins which are characterized by a large hydrophobic domain of 40 or more amino acids. So far the inclusion membrane localization of 22 of them has been confirmed [20] and only a few of them have established functions [18]. IncA is involved in the homotypic fusion of the *C. trachomatis* inclusions [21], [22]. IncB, CT101, CT222 and CT850 co-localized with activated Fyn and Src kinases in inclusion membrane microdomains. These microdomains also interact with the centrosomes and it has been proposed that these four inclusion proteins are involved in the interaction of the inclusion with the microtubule network [23]. CT229 and CT813 have been assigned putative functions in intercepting the host vesicular trafficking, based on their respective interaction with the host proteins Rab4 [24] and VAMP7-8 [25].

Our knowledge of the cellular processes that are targeted by *Chlamydia* has greatly increased over the past 10 years, but we have only begun to identify the host and bacterial factors required for bacterial development. To better understand the molecular mechanisms underlying *C. trachomatis* infection, we recently conducted an RNAi screen and identified CERT as a host factor involved in *C. trachomatis* infection (I.D. and H.A., unpublished). In non-infected cells, CERT is proposed to be involved in the non-vesicular transfer of ceramide at ER-Golgi membrane contact sites (MCSs). Our results indicate that ER-Inclusion MCSs are formed in *C. trachomatis* infected cells. We propose a model in which, through IncD-dependent recruitment of CERT to the inclusion, *C. trachomatis* exploit the non-vesicular lipid transport machinery of the host cell and generate platforms specialized in metabolism and/or signaling events favorable to its replication and development.

## Results

### CERT and VAPB localize to *C. trachomatis* inclusion

We recently conducted an RNAi screen and identified CERT as a host factor involved in *C. trachomatis* infection (I.D. and H.A., unpublished). CERT is proposed to be a functional component of ER-Golgi membrane contact sites (MCSs) (i.e. zone of close apposition (10–50 nm) between two organelles [26], [27]) involved in the non-vesicular transfer of ceramide from the ER to the Golgi [28]. In addition to the carboxy-terminal START domain [29] that binds ceramide, the ER-to-Golgi transfer process requires a central FFAT domain [30] which binds the ER resident proteins VAPA and VAPB (Vesicle-associated membrane protein-associated protein) [31] and an amino-terminal PH domain [32] which binds PI4P and Arf1 on the Golgi membrane [33], [34].

**Figure 1:**
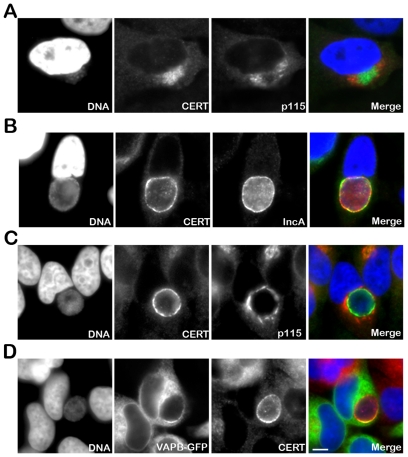
CERT and VAPB localize to *C. trachomatis* inclusion. (A–C) HeLa cells were infected with *C. trachomatis* at a high MOI for 8 h to generate a cluster of incoming bacteria in the perinuclear area of the cell (A) or at a low MOI for 24 h to generate an inclusion originating from a single bacterium (B–C). The infected cells were labeled with antibodies against CERT (CERT, green) (A–C) and the Golgi maker p115 (p115, red) (A, C) or the inclusion membrane protein IncA (IncA, red) (B). (D) HeLa cells expressing a VAPB-GFP fusion protein (VAPB-GFP, green) and infected with *C. trachomatis* for 24 h, were labeled with antibodies against CERT (CERT, red). The host cell nuclei and the bacterial DNA were labeled with the DNA dye Hoechst (DNA, blue) (A–D). The merge images are shown on the right. Scale Bar, 10 µm.

To further investigate the role of CERT in *C. trachomatis* infection, we first determined its cellular localization upon infection. In uninfected cells, the endogenous CERT protein was detected at the Golgi (Supplementary Figure S1). In *C. trachomatis* infected cells, the endogenous CERT protein was highly recruited to *C. trachomatis* inclusion as early as 8 h post infection when incoming bacteria had reached the perinuclear area of the host cells (Figure 1A). As the infection progressed, the inclusion remained CERT positive (Figure 1B) and CERT appeared to localize to the inclusion membrane as shown by co-immuno-staining with the inclusion membrane protein, IncA [35], [36] (Figure 1B). Co-immuno-staining of *C. trachomatis* infected cells with antibodies against CERT and the Golgi markers p115, GM130 or TGN46, did not reveal any co-localization of the two proteins (Figure 1A and 1C and Supplementary Figure S1), showing that the CERT signal did not correspond to the Golgi ministacks surrounding the inclusion in infected cells [9]. However, the CERT signal detected at the inclusion membrane partly overlapped with the ER resident proteins VAPA (data not shown) and VAPB (Figure 1D). These results showed that the lipid transfer protein, CERT, and the ER resident proteins VAPA/B localize to *C. trachomatis* inclusion, suggesting a the close apposition of the ER and the inclusion membrane.

### CERT- and VAPB-positive ER tubules are in close apposition with *C. trachomatis* inclusion

Localization of the ER resident proteins VAPA and VAPB at the inclusion membrane and the previous report of ER tracks containing *Chlamydia* antigens in the vicinity of the inclusion membrane [10] led us to further investigate the potential interaction between the ER and *C. trachomatis* inclusion at the ultra structural level.

**Figure 2:**
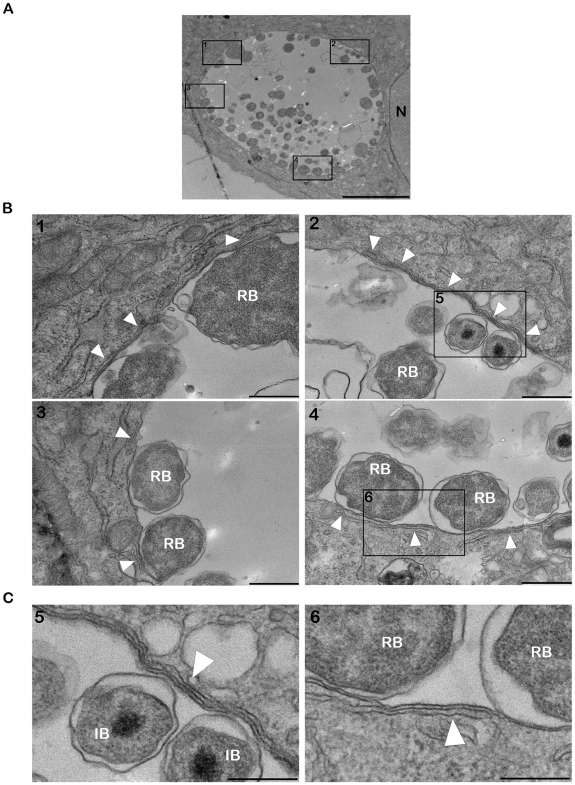
ER tubules are in close apposition with *C. trachomatis* inclusion membrane. (A–C) Electron micrographs of HeLa cells infected with *C. trachomatis* for 24 h. (A) Low magnification micrograph of the section of an entire inclusion. The black boxes numbered 1 to 4 indicate parts of the inclusion that make close contact with the ER tubules. (B) Higher magnification micrographs corresponding to the black boxes numbered 1 to 4 in (A). (C) Close up of the black boxes numbered 5 and 6 in (B) showing the close apposition (∼10 nm) of the ER tubules with the inclusion membrane. N, Nucleus. RB, *Chlamydia* Reticulate Body. IB, *Chlamydia* Intermediate Body. White arrowheads, ER tubules. Scale bar, 5 µm (A), 500 nm (B), 250 nm (C).

Electron microscopy analysis of *C. trachomatis* infected cells revealed the presence of ER tubules in close proximity of the inclusion membrane (Figure 2). On the inclusion section depicted in Figure 2, the ER tubules covered ∼25% of the inclusion membrane (Figure 2A and 2B). The longest tubule was ∼2 µm long (Figure 2B, panel 2) and the shortest tubules were ∼250 nm (Figure 2B, panel 3). Although, the number (1–6) and the size of the tubules (∼250 nm to 2 µm) varied among the inclusion sections analyzed, all inclusions were to some extent associated with ER tubules, suggesting a substantial coverage of the inclusion membrane. The distance between the ER tubules and the inclusion membrane was ∼10 nm (Figure 2C, panels 5 and 6).

**Figure 3:**
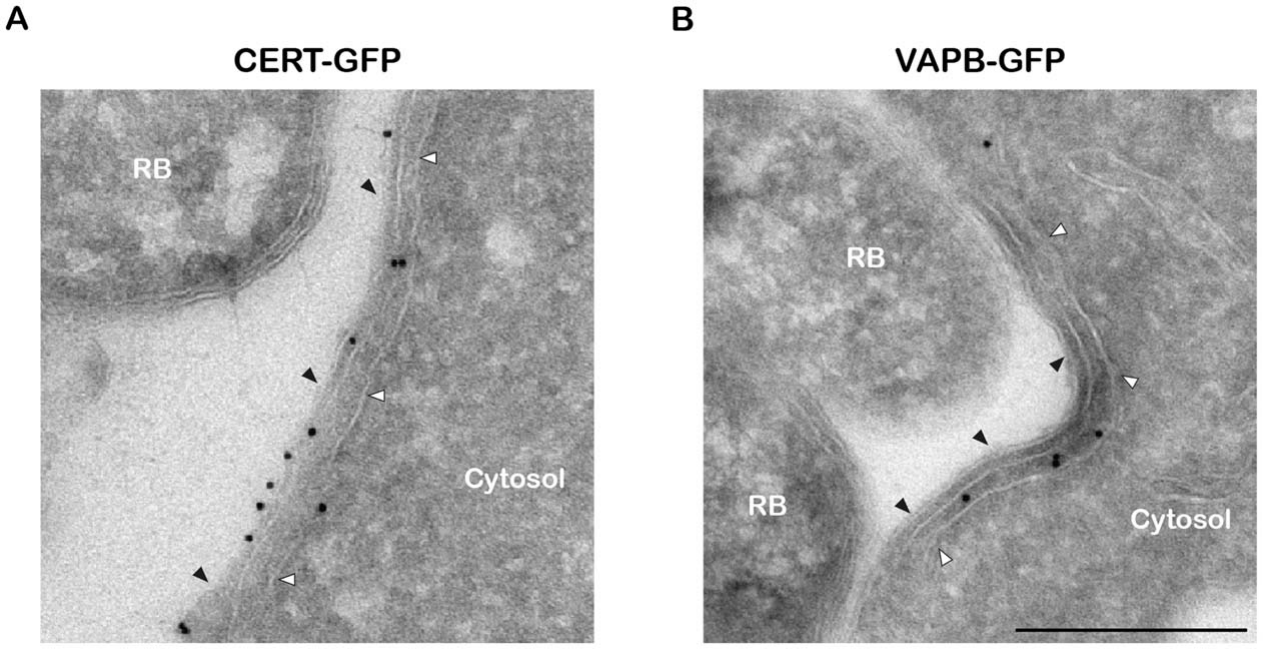
CERT- and VAPB-positive ER tubules are in close apposition with *C. trachomatis* inclusion. (A–B) Cryo-immunogold electron micrographs of HeLa cells expressing CERT-GFP (A) or VAPB-GFP (B) fusion proteins and infected with *C. trachomatis* for 24 h. The sections were labeled with anti-GFP antibodies coupled to 10 nm gold particles. RB, *Chlamydia* Reticulate Body. Black arrowheads, Inclusion membrane. White arrowheads, ER tubule. Scale Bar, 250 nm.

We next investigated the cellular localization of CERT and VAPB with regard to the inclusion membrane and/or the ER tubules. Cryo-electron microscopy analysis confirmed the presence of ER tubules in close proximity (<20 nm) of the inclusion membrane (Figure 3A and 3B). Immunogold labeling showed that CERT localized to the inclusion membrane and was enriched at points of contact with the ER tubules (Figure 3A). VAPB was not observed on the inclusion membrane, but located to the ER tubules observed in close proximity of the inclusion (Figure 3B).

Altogether these results are consistent with the notion that, similar to the ER-Golgi MCSs observed in non-infected cells [26], [27], ER-Inclusion MCSs are established upon *C. trachomatis* infection and the non-vesicular ceramide transfer protein CERT and the ER resident proteins VAPB localize to these points of contact.

### The PH domain of CERT mediates its recruitment to the inclusion

**Figure 4:**
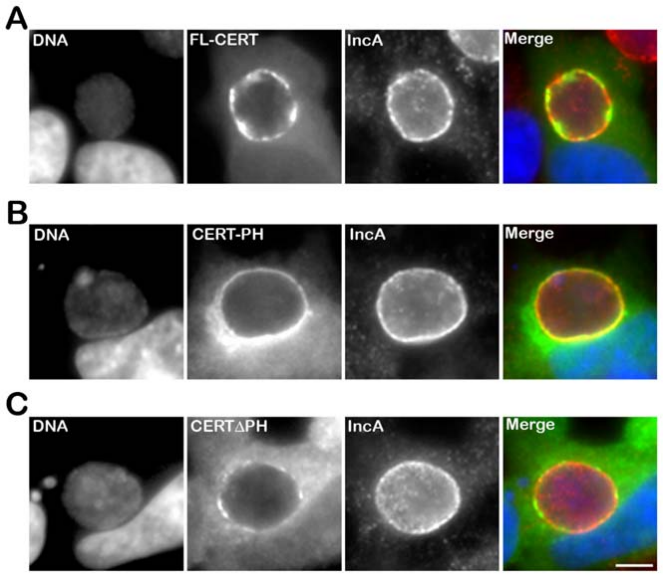
The PH domain mediates the recruitment of CERT to the inclusion. HeLa cells expressing CERT-GFP fusion proteins (green), containing either full-length CERT (FL-CERT) (A) or the PH domain of CERT only (CERT-PH) (B) or CERT deleted of its PH domain (CERT ΔPH) (C), and infected with *C. trachomatis* for 24 h, were labeled with the inclusion membrane protein IncA (IncA, red). The DNA dye Hoechst labeled the host cell nuclei and the bacterial DNA (DNA, blue). The merge images are shown on the right. Scale Bar, 10 µm.

We next determined the CERT domain required for its recruitment to the inclusion membrane. As observed with the endogenous CERT (Figure 1), a CERT-GFP fusion protein containing the full length CERT was recruited to *C. trachomatis* inclusion membrane (Figure 4A, FL-CERT and Supplementary Figure S2, FL-CERT CTRLsiRNA). Similarly, a construct containing the PH domain only was also recruited to *C. trachomatis* inclusion (Figure 4B, CERT-PH). However, a CERT construct lacking the PH domain was mostly cytosolic and, except for very small patches, was no longer recruited to the inclusion membrane (Figure 4C, CERTΔPH and Supplementary Figure S2 CERTΔPH CTRLsiRNA). It is likely that, in these experiments, ER-Inclusion MCSs were formed. Thus, remaining patches observed in Figure 4C (and Supplementary Figure S2) with the CERTΔPH-GFP construct probably reflected binding of the fusion protein to the endogenous VAPs through its FFAT domain. Accordingly, the number of patches was further decreased in VAPA/B-depleted cells (Supplementary Figure S2 CERTΔPH VAPA&BsiRNA). Altogether, these results indicate that the PH domain of CERT is necessary and sufficient for CERT association with *C. trachomatis* inclusion membrane.

### CERT recruitment to the inclusion is Arf1-independent

Our results suggested that, at ER-Inclusion MCSs, CERT interacted with the ER tubules by binding to the VAPs, and with the inclusion membrane *via* its PH domain. The interacting partner(s) on the inclusion membrane remained however to be identified. CERT belongs to a family of lipid transfer proteins, containing OSBP and FAPP [33], [37]. These proteins are characterized by a common amino-terminal PH domain, which governs their specific association with Golgi membranes. For OSBP and FAPP, this specificity is conferred by the recognition and binding of the PH domain to PI4P and Arf1. Both determinants are simultaneously required since reduced level of PI4P onto Golgi membrane or Arf1 inactivation reduces the Golgi association of OSBP and FAPP PH domains [38], [39]. Association of CERT PH domain with Golgi membranes also depends on PI4P [28], [40]. By analogy with OSBP and FAPP, it has been proposed that Arf1 might also control CERT association with Golgi membranes [33].

**Figure 5:**
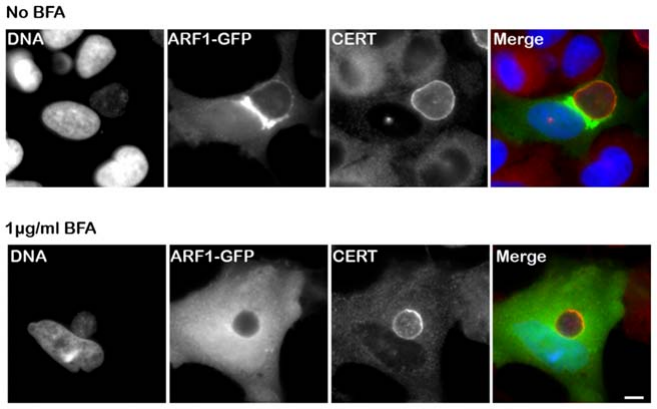
CERT recruitment to the inclusion is ARF1-independent. HeLa cells expressing Arf1-GFP (ARF1, green) were infected for 24 h with *C. trachomatis* in the absence (upper panels) or in the presence of 1 µg/ml BFA (lower panels) and labeled using antibodies against CERT (red). The DNA dye Hoechst labeled both the nuclei and *C. trachomatis* (DNA, blue). The merge images are shown on the right. Scale Bar, 10 µm. *C. trachomatis* infection, performed in the presence of BFA, led to the formation of smaller inclusions as previously reported by Hackstadt *et. al.* [12].

Because Arf1 is present onto the inclusion membrane [41] (Figure 5, top panels, Arf1-GFP), we tested the functional importance of Arf1 with respect to CERT recruitment to the inclusion. As expected, Brefeldin A treatment abolished Arf1 interaction with the inclusion [41] (Figure 5, bottom panels, Arf1-GFP). However, it did not affect endogenous CERT recruitment to *C. trachomatis* inclusion (Figure 5, bottom panels, CERT). We observed a similar result in Arf1-depleted cells (not shown). These results suggested that the PH domain of CERT mediates its recruitment to *C. trachomatis* inclusion through an Arf1-independent mechanism.

### The *Chlamydia* inclusion protein IncD specifically interacts with the PH domain of CERT

To identify the determinants underlying the recruitment of CERT to the *C. trachomatis* inclusion, we performed immuno-precipitation experiments with extracts obtained from cells expressing a FLAG-tagged version of CERT. Comparative mass-spectrometry analyses of uninfected and infected samples led to the identification of the *C. trachomatis* effector protein IncD as a potential binding partner for CERT (see Methods for details and Supplementary Figure S3 for *C. trachomatis* replication in HEK293 cells).

**Figure 6:**
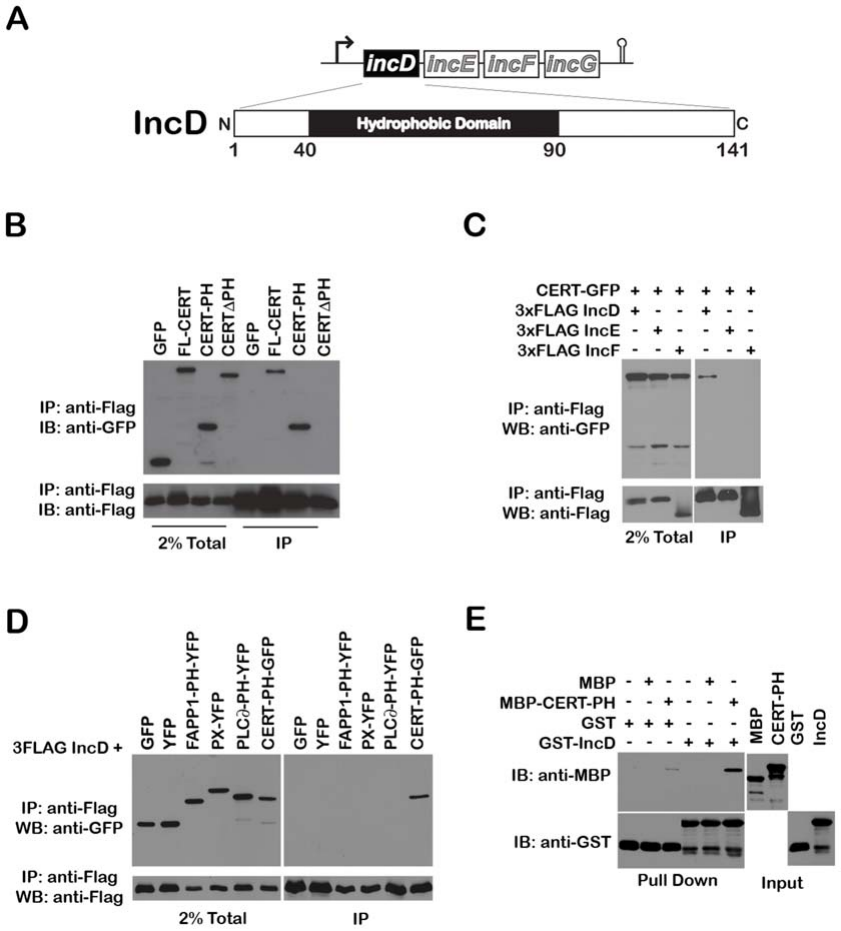
The *Chlamydia* inclusion protein, IncD, interacts with the PH domain of CERT. (A) Schematic representation of the *incDEFG* operon and the IncD protein. (B) Lysates from HEK293 cells co-expressing 3xFLAG-IncD and the indicated GFP-CERT fusion proteins were immunoprecipitated with anti-FLAG M2 beads. A portion of the cell lysate (Left Panel, 2% Total) and the immunoprecipitated proteins (Right Panel, IP) were separated by SDS-PAGE and analyzed by immunoblot with antibodies against GFP (Top Panels) and FLAG (Bottom Panels). (C) Lysates from HEK293 cells co-expressing 3xFLAG-IncD, 3xFLAG-IncE or 3xFLAG-IncF and GFP-CERT fusion proteins were immunoprecipitated with anti-FLAG M2 beads. A portion of the cell lysate (Right Panel, 2% Total) and the immunoprecipitated proteins (Left Panel, IP) were separated by SDS-PAGE and analyzed by immunoblot with antibodies against GFP (Top Panels) and FLAG (Bottom Panels). (D) Lysates from HEK293 cells co-expressing 3xFLAG-IncD and the indicated GFP or YFP fusion proteins were immunoprecipitated with anti-FLAG M2 beads. A portion of the cell lysate (Right Panel, 2% Total) and the immunoprecipitated proteins (Left Panel, IP) were separated by SDS-PAGE and analyzed by immunoblot with antibodies against GFP (Top Panels) and FLAG (Bottom Panels). (E) The indicated GST fusion proteins, immobilized onto glutathione sepharose were incubated in the presence of the indicated purified MBP fusion proteins. The protein complexes bound to the resin were separated by SDS-PAGE and analyzed by immunoblot with antibodies against MBP (Top Panels) and GST (Bottom Panels). The inputs for each purified protein are shown in the right panels (Input).

IncD is the product of the first gene of an operon containing three other inclusion proteins (IncE, IncF and IncG) [42] and displays a large central hydrophobic domain [42], [43] (Figure 6A). The IncD-G operon is expressed within the first two hours of *C. trachomatis* developmental cycle and all four proteins were shown to localize to the inclusion membrane [42]. Both spatial and temporal expression of IncD made it an attractive candidate for CERT interaction.

We tested the specificity of CERT/IncD interaction by co-immuno-precipitation experiments. While a CERT-GFP construct (but not GFP alone) co-immuno-precipitated with the FLAG-tagged version of IncD (Figure 6B, IP, lanes 1–2), the FLAG-tagged versions of IncE and IncF, two Inc proteins that had a cellular localization similar to IncD when over-expressed in eukaryotic cells (i.e. the ER) (Supplementary Figure S4), did not (Figure 6C). We next confirmed that CERT/IncD interaction is mediated through the PH domain of CERT. As expected, a FLAG-tagged version of IncD co-immunoprecipitated with a CERT-PH-GFP fusion protein, but did not co-immunoprecipitate with a CERTΔPH-GFP construct lacking the PH domain (Figure 6B, IP, Lanes 3–4). We confirmed the specificity of IncD interaction with the PH domain of CERT by showing that the FLAG-tagged version of IncD failed to co-immunoprecipitate the phosphoinositide binding domain of FAPP1 [38], p40phox (PX) [44] or PLC∂ [45] (Figure 6D). Finally, we confirmed the direct interaction between IncD and the PH domain of CERT by *in vitro* binding assay, using CERT PH domain and IncD purified as MBP and GST fusion proteins, respectively. As shown in Figure 6E, the PH domain of CERT interacted strongly with GST-IncD fusion protein, but not with GST alone. Altogether, these experiments demonstrated that the *C. trachomatis* inclusion membrane protein IncD binds specifically to the PH domain of CERT, suggesting that IncD is involved in CERT recruitment to the *C. trachomatis* inclusion.

### IncD co-localizes with CERT onto *C. trachomatis* inclusion membrane

**Figure 7:**
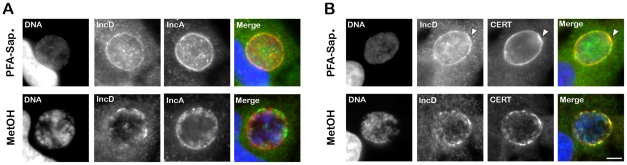
IncD co-localizes with CERT onto *C. trachomatis* inclusion membrane. (A–B) HeLa cells infected with *C. trachomatis* for 24 h were fixed and permeabilized using 4% PFA and Saponin, respectively, (PFA-Sap., top panels) or Methanol (MetOH, bottom panels) and labeled with antibodies against the inclusion membrane protein IncD (IncD, green) (A–B) and the inclusion membrane protein IncA (IncA, red) (A) or CERT (CERT, red) (B). The host cell nuclei and the bacterial DNA were labeled with the DNA dye Hoechst (DNA, blue). The merge images are shown on the right. The arrowheads indicate an area of the inclusion membrane enriched in CERT and IncD. Scale Bar, 10 µm.

We next investigated IncD localization onto *C. trachomatis* inclusion membrane. When *C. trachomatis* infected cells, were fixed and permeabilized using paraformaldehyde and saponin, respectively, IncD displayed inclusion membrane localization (Figure 7A, top panels). However, not all inclusions were IncD positive, although they were all positive for IncA (Supplementary Figure S5), suggesting that the IncD antigen was not efficiently revealed. When the cells were fixed and permeabilized using methanol, all inclusions were positive for IncA and IncD (Figure 7A, bottom panels).

We next investigated whether IncD co-localized with CERT onto the inclusion membrane. When *C. trachomatis* infected cells were fixed and permeabilized using paraformaldehyde and saponin, respectively, both markers localized to the inclusion membrane (Figure 7B, top panels). As previously observed (Figure 1), the CERT signal was not homogeneous and appeared more intense in some areas of the inclusion membrane, some of which also appeared enriched in IncD (Figure 7B, top panels, arrowhead). When the cells were fixed with methanol (Figure 7B, bottom panels), CERT localization was patchier than in paraformaldehyde-fixed cells and all patches were positive for both CERT and IncD. Altogether, these results suggested that IncD and CERT localize to the inclusion membrane and may be enriched/stabilized at specific macrodomains of the inclusion membrane.

### CERT and VAPA/B depletion impairs *C. trachomatis* inclusion development

**Figure 8:**
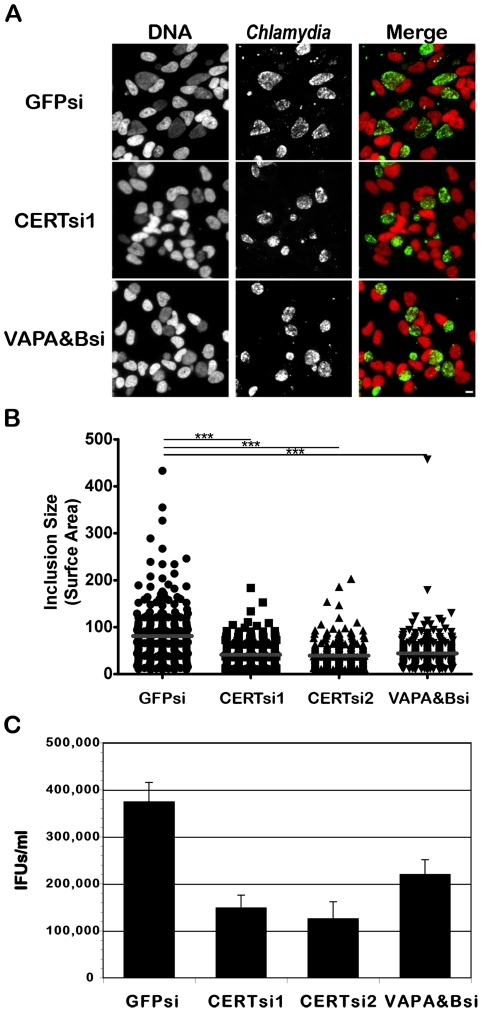
CERT and VAPA/B depletion impairs *C. trachomatis* inclusion development. HeLa cells were transfected with control siRNA (GFPsi) or two different pool of CERT siRNA (CERTsi1 and CERTsi2) or a pool of siRNA against VAPA and VAPB (VAPA&Bsi) for 3 days and infected with *C. trachomatis* for 32 h. (A) Immunofluorescence images to illustrate the difference in inclusion size between control (GFPsi) and CERT- (CERTsi1) or VAPA/B- (VAPA&Bsi) depleted cells. The cells were fixed and labeled with an antibody against *C. trachomatis* (*Chlamydia*, green). The DNA dye Hoechst labeled the host cell nuclei and the bacterial DNA (DNA, red). The merge images are shown on the right. Scale Bar, 10 µm. (B) For each condition, the surface area of 300 inclusions was determined. Each point represents data from a single inclusion. The grey lines indicate the mean values from the data for each condition. The difference between control and CERT or VAPA&B siRNA was statistically significant; ***P<0.0001 (Student's t test). (C) The number of infectious bacteria measured as IFUs/ml was determined 48 h p.i.. Data show the mean and standard deviation of triplicates of a representative experiment.

To demonstrate the physiological relevance of CERT and VAPA/B recruitment to the *C. trachomatis* inclusion, we depleted CERT using two independent pools of CERT siRNA duplexes and VAPA and VAPB using a combination of pools of VAPA and VAPB siRNA duplexes. Transfection of the siRNA duplexes resulted in efficient depletion of all proteins (see Supplementary Figure S6 for CERT, VAPA and VAPB knock-down efficacy at the mRNA and protein level and Supplementary Table S1 for siRNA duplex sequences). In addition, CERT was no longer detected on the surface of *C. trachomatis* inclusion in CERT depleted cells (Supplementary Figure S7). Importantly, these experiments revealed that *C. trachomatis* inclusions were smaller in CERT-depleted cells as compared to control siRNA-treated cells (Figure 8A and 8B). The overall reduction in inclusion size upon CERT depletion also correlated with a reduction in infectious progeny (Figure 8C). Similar to the situation observed with CERT, depletion of VAPA and VAPB led to the formation of smaller inclusions (Figure 8A and 8B) and the number of infectious progeny produced was also reduced (Figure 8C). These experiments confirmed a role for CERT and VAPA/B in *C. trachomatis* development.

## Discussion

### CERT and VAPA/B recruitment to *C. trachomatis* inclusion

We showed that the lipid transfer protein, CERT, was recruited to *C. trachomatis* inclusion (Figure 1 and Figure 4A). The CERT signal was not evenly distributed onto the inclusion and appeared more concentrated in some areas. Although CERT localizes to Golgi membranes in uninfected cells, the CERT signal observed upon infection did not correspond to the Golgi ministacks surrounding the inclusion in infected cells [9], but rather corresponded to the inclusion membrane. These data indicate that CERT is enriched in some macrodomains of the inclusion membrane.

Our results also showed that the ER resident proteins, VAPA and VAPB, were recruited to *C. trachomatis* inclusion in areas enriched in CERT (Figure 1). VAPB however did not localized to the inclusion membrane but rather to ER tubules that were in close proximity of the inclusion membrane (Figure 3), suggesting a potential interaction between the ER and the inclusion.

### ER-Inclusion MCSs

Many cellular organelles have been shown in close apposition to or interacting with the *Chlamydia* inclusion, including the Golgi [9], mitochondria [46], multivesicular bodies [13] and lipid droplets [17]. Tracks of ER have also been reported in the proximity of *C. trachomatis* inclusion [10]. Our electron microscopy analysis (Figure 2 and Figure 3) is in agreement with this latest observation, since it revealed the close apposition of ER tubules to *C. trachomatis* inclusion and therefore suggests a direct interaction between the ER and the inclusion through the formation of ER-Inclusion MCSs.

MCSs are defined as a zone of close apposition (10–50 nm) between two organelles. In eukaryotes, the ER has been described as one of the partnering organelle and has been shown to make close contact with the yeast vacuole, the plasma membrane, the Golgi complex, endosomes and lysosomes, and mitochondria. Except for the yeast ER/Vacuole MCSs [47], bridging complexes, also referred to as structural components, that bring the two partnering membranes in close apposition and stabilize of MCSs, have not been identified. In contrast, an increasing number of proteins known to function on two contacting organelles have been identified and are referred as functional components [26], [27], including CERT, which is proposed to be a functional component of ER-Golgi MCSs.

The distance between the ER tubules and *C. trachomatis* inclusion membrane was 10–20 nm (Figure 2 and Figure 3), which is in agreement with the notion of ER-Inclusion MCSs. We cannot exclude that, as described for the yeast ER/Vacuole MCSs [47], specific structural components are involved in the formation and maintenance of the ER-Inclusion MCSs. These factors could be of mammalian and/or bacterial origin. Our results however indicate that, CERT, a previously proposed functional component of ER-Golgi MCSs, is probably also a functional component of ER-Inclusion MCSs.

### Role of Arf1 and PI4P in CERT association with *C. trachomatis* inclusion membrane

CERT belongs to a family of lipid transfer proteins, containing OSBP and FAPP [33], [37]. These proteins are characterized by a common amino-terminal PH domain, which governs their specific association with Golgi membranes by recognizing and binding to PI4P (OSBP, FAPP and CERT) and Arf1 (OSBP and CERT). By analogy with OSBP and FAPP, it has been proposed that Arf1 might also control CERT association with Golgi membranes [33]. Because Arf1 and PI4P are also present at the inclusion membrane [41], it made them likely components involved in CERT recruitment to *C. trachomatis* inclusion membrane.

Moorhead *et. al.* study showed that OSBP-PH and CERT-PH (referred as GPBP in this study) associated with *Chlamydia* inclusion membrane. However, the association was not sensitive to Brefeldin A, suggesting that it was Arf1-independent. In agreement, we showed that Brefeldin A or Arf1 depletion did not affect endogenous CERT association with the inclusion membrane (Figure 5), confirming that Arf1 is not involved in this process.

Moorhead *et. al.* showed that the association of OSBP-PH with the inclusion membrane was however sensitive to a mutation in OSBP-PH domain that abolished PI4P binding (but not Arf1 binding). This result led the author to conclude that the localization of PI4P-binding PH domains to the inclusion reflected the presence of PI4P at the inclusion membrane. We have attempted to confirm the involvement of PI4P in the endogenous CERT association with the inclusion by depleting enzyme involved in PI4P synthesis. PI4KIIIß has been identified as the main source of PI4P on Golgi membranes and therefore a key regulator of CERT, OSBP and FAPP association with Golgi membrane [33], [37]. Moorhead *et. al.* have proposed that OCRL1 and PI4KIIα might contribute to the pool of *Chlamydia* inclusion membrane PI4P [41]. Treatment with a specific PI4KIIIß inhibitor (PIK93) or depletion of PI4KIIIß, OCRL1 or PI4KIIα proteins had no effect on CERT recruitment to *C. trachomatis* inclusion (not shown). Altogether, these results left open the role of PI4P in CERT association with the *C. trachomatis* inclusion membrane.

### Role of IncD in CERT recruitment to the inclusion membrane

We have identified the *C. trachomatis* inclusion membrane protein IncD as an *in vivo* binding partner of CERT and we showed that IncD interacted with the PH domain of CERT (Figure 6). This interaction was highly specific because IncD did not interact with other phosphoinositide-binding domain and CERT did not interact with other Inc proteins (i.e. IncE or IncF). Our results therefore suggested a role for IncD in the association of CERT with the inclusion membrane.

In agreement with this notion, both IncD and CERT localized to *C. trachomatis* inclusion membrane (Figure 7). Various methods of fixation suggested that IncD and CERT displayed partial co-localization into patches. In addition, CERT appeared enriched in some areas that were in contact with VAPB positive tubules (Figure 1). These data suggest that the enrichment of CERT and IncD onto macrodomains of the inclusion membrane may correspond to the point of contacts with the ER where IncD/CERT/VAPB form a complex through the binding of CERT to IncD onto the inclusion membrane and to VAPB onto the ER tubules. Further characterization of IncD/CERT/VAPB interaction will be required to identify the determinants that drive the specific formation of this complex.

Altogether, our results suggest that *C. trachomatis* has evolved strategies to efficiently hijack CERT and relocate it to the inclusion membrane. Unfortunately, because genetic tools are not available to manipulate *Chlamydia*, we were not able to assay whether a *C. trachomatis* IncD mutant is impaired in CERT localization to the inclusion and whether its developmental cycle is perturbed. We were however able to show that *C. caviae*, a strain of *Chlamydia* that infect guinea pigs and that is lacking IncD, was not able to recruit CERT-GFP to its inclusion membrane (Supplementary Figure S8), confirming the specific role of IncD in CERT recruitment to the inclusion.

Although our *in vitro* binding study demonstrated that IncD interact with the PH domain of CERT in the absence of additional factors (Figure 6), we cannot exclude the involvement of additional host or *C. trachomatis* factors *in vivo*, including PI4P. If PI4P plays a role in CERT association with the inclusion membrane, it would be interesting to determine whether the analogy could be made between PI4P/Arf1 on Golgi membranes and PI4P/IncD on *C. trachomatis* inclusion membrane. On the other hand, if PI4P is not required for CERT association with *C. trachomatis* inclusion membrane, it would suggest that IncD may mimic the Arf1/PI4P determinants that usually drive CERT recruitment to Golgi membranes. We also note that IncD could act in concert with some yet to be discovered host and/or bacterial factors that localize at the inclusion membrane. Further structure/function analyses of IncD will be required to address these questions.

### CERT and VAPA/B depletion and *Chlamydia* development

Our results showed that CERT or VAPA/B depletion was detrimental to *C. trachomatis* development (Figure 8), but the specific role of these proteins remains to be determined. Previous studies have shown that sphingomyelin-containing vesicles traffic from the Golgi ministacks that surround the inclusion, to the inclusion, where sphingomyelin is incorporated into the inclusion membrane as well as the cell wall of the bacteria. It was also shown that acquisition of sphingomyelin is essential for *Chlamydia* development [9], [12], [48]–[50]. Since CERT is involved in the non-vesicular trafficking of the sphingomyelin precursor ceramide from the ER to the Golgi, an overall defect in sphingomyelin synthesis could explain the defect in *C. trachomatis* development observed in CERT-depleted cells.

Although we did not measure sphingomyelin synthesis in CERT siRNA-treated cells, studies from the Hanada group using the LY-A cell line (in which CERT is inactive due to a mutation in the PH domain that prevents CERT association with Golgi membranes) and fluorescent ceramide or [^3^H]sphingosine, revealed that the LY-A cell line showed at least a 50% reduction in sphingomyelin biosynthesis [28], [51], supporting the hypothesis that CERT siRNA-treated cells might have reduced level of sphingomyelin. These studies however also suggested the existence of a CERT-independent pathway for sphingomyelin synthesis that could account for up to 50% of sphingomyelin synthesis.

We did not observe any major disruption of the Golgi morphology in CERT- or VAPA/B-depleted cells (Supplementary Figure S9) and 60% of the inclusions had detectable Golgi ministacks around them in both control and CERT depleted cells, suggesting that the Golgi fragmentation necessary for optimal sphingomyelin acquisition was not impaired. When infected cells were labeled with fluorescent ceramide, which is subsequently metabolized into sphingomyelin in the Golgi, and incorporated into the inclusion [12], [48], [49], we observed the accumulation of fluorescent lipids at the Golgi and in *C. trachomatis* inclusion from control or CERT- or VAPA/B-depleted cells (Supplementary Figure S10, S11, S12 and S13). These results suggested that lipids were still trafficked to the inclusion in CERT siRNA-treated cells and it is most likely that, in the context of CERT siRNA-treated cells, the CERT-independent pathway of ceramide trafficking from the ER to the Golgi, explain the labeling of the Golgi. Labeling of the inclusion could then occur through vesicular trafficking of sphingomyelin from the Golgi as previously described [12], [48], [49].

If both CERT-dependent and –independent pathways participate to the accumulation of fluorescent lipids in the inclusion, one could expect a reduction in fluorescence in CERT siRNA-treated cells. The rapid bleaching of the fluorescent signal of BODIPY-C5-Ceramide, however did not allow us to accurately quantify the fluorescence intensity of the inclusions. Another limitation of this assay is that it does not determine the nature of the fluorescent lipid(s) that accumulate in the inclusion. Additional experiments involving lipid extraction and analysis will be required to unambiguously determine the nature of the fluorescent lipid(s) that we observed in the inclusion in CERT- VAPA/B siRNA-treated cells.

Altogether, it is possible that a partial overall reduction in host cell sphingomyelin explains the partial reduction in *C. trachomatis* growth observed in CERT siRNA treated cells, however further experiments, including the uncoupling of CERT-dependent and –independent pathways, are required to unequivocally address this question.

Given the localization of CERT to the inclusion, the described role of CERT in ceramide transfer and the presence of ER tubules (source of ceramide) in close apposition with the inclusion membrane, we favor the idea that CERT transfers ceramide from the ER to the inclusion membrane. Because of the requirement of *Chlamydia* for sphingomyelin for replication, ceramide could serve as a precursor for sphingomyelin synthesis directly at the inclusion membrane, assuming that a sphingomyelin synthase of host or bacterial origin is also present at the inclusion membrane. Alternately, it is possible that ceramide accumulates at the inclusion without being further modified and serves as a signaling molecule at the inclusion membrane. For example, it has been proposed that the lipid transfer/binding proteins (LT/BPs) Nir2 [52] and OSBP [53], which are respectively involved in the non-vesicular transfer of phosphatidylinositol/phosphatidylcholine (PI/PC) and sterols at ER-Golgi MCSs, act together with CERT to affect the lipid composition of the Golgi membranes and therefore influence the structural and functional identities of these membranes [54]. Similarly, the CERT-dependent transfer of ceramide at ER-Inclusion MCSs could influence the lipid composition of the inclusion membrane and generate specialized metabolic and/or signaling microenvironment favorable for bacterial development. Whether other LT/BPs, such as Nir2 or OSBP, or additional host factors localize to ER-Inclusion MCSs and participate in the formation of these specialized platforms remain to be determined. Finally, we cannot exclude that CERT has a yet to be discovered role at the inclusion membrane that does not involve ceramide transport.

### Conclusion

**Figure 9:**
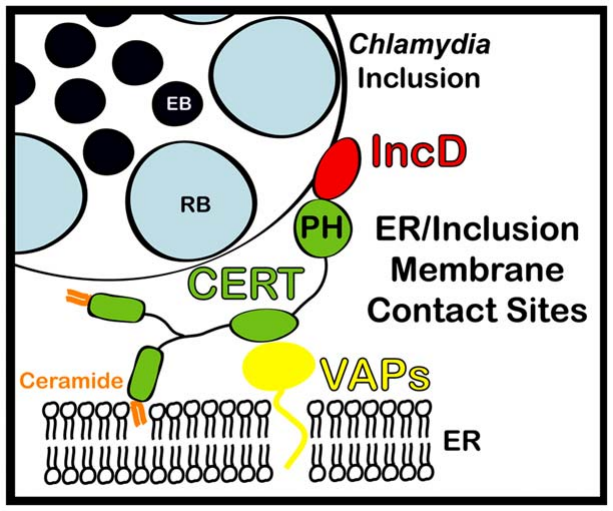
Schematic representation of ER-*Chlamydia* inclusion membrane contact sites. ER-Inclusion membrane contact sites are formed upon *Chlamydia* infection. These sites are enriched for the ceramide transfer protein CERT. CERT contacts the ER by binding to VAPA/VAPB and interacts with IncD onto the inclusion membrane, through its PH domain. The close apposition of ER tubules to the inclusion membrane may produce a dynamic environment specialized in non-vesicular trafficking of lipids, such as ceramide, leading to metabolism and signaling events that will ensure proper bacterial development.

Altogether our results suggest a model in which the *C. trachomatis* effector protein IncD specifically interacts with the non-vesicular ceramide transfer protein CERT, at MCSs between *C. trachomatis* inclusion membrane and ER tubules harboring the VAPA/B proteins (Figure 9). We speculate that the IncD-CERT-VAPA/B interaction may be involved in the non-vesicular transfer of ceramide from the ER to the inclusion. We however cannot exclude a more complex role for ER-Inclusion MCSs in supporting bacterial development. Further studies of the ER-Inclusion MCSs, including the identification of additional structural and functional components, may not only reveal the mechanisms underlying *C. trachomatis* pathogenesis, but may also illuminate the poorly understood cellular mechanisms underlying inter-organelle communication.

## Materials and Methods

### Cell lines and bacterial strains

HeLa cells and HEK293 cells (ATCC) and HeLa229 cells (Dautry-Varsat Laboratory, Pasteur Institute, Paris, France) were cultured at 37°C with 5% CO_2_ in DMEM high glucose (Invitrogen) supplemented with 10% heat inactivated FBS (Invitrogen). *C. trachomatis Lymphogranuloma venereum, Type II* were obtained from ATCC (L2/434/Bu VR-902B). *C. caviae* was obtained from the Dautry-Varsat Laboratory (Pasteur Institute, Paris, France) and was originally from Roger Rank Laboratory (Little Rock, Arkansas). *Chlamydia* propagation and infection was performed as previously described [55].

### siRNA and DNA transfection

The protocol used for siRNA transfection was adapted from Dharmacon HeLa cells transfection's protocol (www.dharmacon.com) [55]. The sequences of the siRNA duplexes used in this study are described in Supplementary Table S1. DNA transfection was performed using Fugene 6 according to the manufacturer recommendations. DNA transfection of siRNA treated cells was performed 2 days post siRNA transfection.

### Quantitative PCR

Total RNA and first-strand cDNA synthesis was performed using the TaqMan Gene Expression Cells-to-Ct Kit (Applied Biosystems) as recommended by the manufacturer with the addition of DNaseI for the removal of unwanted genomic DNA. mRNA levels were determined by quantitative real-time PCR using the LightCycler 480 Master Kit and LightCycler 480 instrument (Roche Biochemicals, Indianapolis, IN). The combination of probes and primers that were used to determine the relative amount of target mRNA by quantitative PCR is described in Supplementary Table S2.

### DNA constructs

The PH domain of CERT, VAPA and VAPB ORFs were amplified from HeLa cell cDNA. The ORF corresponding to full length CERT was amplified from a myc-CERT construct [56]. IncD, IncE and IncF ORFs were amplified from *C. trachomatis* genomic DNA. The primers and vectors used in this study are described in Supplementary Table S3. Arf1-GFP [57] and PX-YFP [44] were described previously.

### Immunoblotting

Protein samples were separated by SDS-PAGE and analyzed by immunoblot using HRP-conjugated secondary antibodies and Amersham ECL western blotting detection reagents.

### Immunofluorescence and microscopy

At the indicated time, the cells seeded onto glass coverslips were fixed for 30 min in PBS containing 4% paraformaldehyde. Immunostaining were performed at room temperature. Antibodies were diluted in PBS containing 0.16 µg/ml Hoechst (Molecular Probes), 0.1% BSA and 0.05% Saponin. Samples were washed with PBS containing 0.05% Saponin and a final PBS wash was performed before examination under an epifluorescence microscope. Samples presented in the lower panels of Figure 7 were fixed in ice-cold methanol for 5 min and labeled with antibodies diluted in PBS containing 0.1% BSA. Images presented in Supplementary Figure 2 were acquired using a confocal microscope.

### Antibodies

The following primary antibodies were used: rabbit polyclonal anti-*C. trachomatis* (1∶300 (IF), Virostat), rabbit polyclonal anti-*C. trachomatis* IncA (1∶200 (IF), kindly provided by T. Hackstadt, Rocky Mountain Laboratories), rabbit polyclonal anti-*C. caviae* IncA (1∶300 (IF), kindly provided by A. Subtil, Pasteur Institute, Paris, France), mouse polyclonal anti-*C. trachomatis* IncD (1∶300 (IF), kindly provided by G. Zhong, University of Texas Health Science Center at San Antonio), chicken polyclonal anti-CERT (1∶200 (IF), 1∶1,000 (WB) Sigma), mouse anti-p115 (1∶200 (IF), kindly provided by G. Waters), mouse anti-GM130 (1∶200 (IF), BD Transduction Lab.), sheep anti-TGN46 (1∶200 (IF), Serotec), rabbit polyclonal anti-GFP (1∶200 (EM), 1∶2,000 (WB), Invitrogen), mouse anti-FLAG (1∶20,000 (WB), Sigma), rabbit polyclonal anti-VAPB (1∶200 (WB), Abcam), rabbit polyclonal anti-actin (1∶10,000 (WB), Sigma).

The following secondary antibodies were used: goat anti-rabbit AlexaFluor 594 antibody (1∶500, Molecular Probes), goat anti-mouse AlexaFluor 594 antibody (1∶500, Molecular Probes), donkey anti-sheep AlexaFluor 594 antibody (1∶500, Molecular Probes), Fluorescein (FITC) donkey anti-chicken IgY antibody (1∶300, Jackson ImmunoResearch), peroxidase-conjugated goat anti-rabbit IgG (1∶10,000, Jackson ImmunoResearch), peroxidase-conjugated goat anti-mouse IgG (1∶10,000, Jackson ImmunoResearch), peroxidase-conjugated donkey anti-chicken IgY (1∶10,000, Pierce).

### Electron microscopy

HeLa cells seeded onto glass coverslips and infected with *C. trachomatis* for 24 h were fixed in 2.5% glutaraldehyde in 0.1 M sodium cacodylate buffer pH 7.4 with 2% sucrose for 1 hour. The samples were rinsed 3 times in sodium cacodylate buffer then were postfixed in 1% osmium tetroxide for 1 hour, en bloc stained in 2% uranyl acetate in maleate buffer pH 5.2 for a further hour then rinsed, dehydrated in an ethanol series and infiltrated with epon resin. The coverslips were then covered with resin filled capsules and baked over night at 60°C. Hardened block enface sections were cut using a Leica UltraCut UCT. 60 nm sections were collected and stained using 2% uranyl acetate and lead citrate. Samples were all viewed FEI Tencai Biotwin TEM at 80 Kv. Images were taken using Morada CCD and iTEM (Olympus) software.

### Immuno electron microscopy

HeLa cells were transfected with a GFP-CERT or GFP-VAPB construct for 18 h and infected with *C. trachomatis* for an additional 24 h. The cells were washed with PBS, fixed in PBS containing 4% Paraformaldehyde/0.1% Glutaraldehyde for 15 min at room temperature, followed by 45 min at 4°C in 4% paraformaldehyde and re-suspended in 10% gelatin. Trimmed smaller blocks were placed in 2.3 M sucrose overnight on a rotor at 4°C. Then transferred to aluminum pins and frozen rapidly in liquid nitrogen. The frozen block were trimmed on a Leica Cryo-EMUC6 UltraCut and 65 nm thick sections were collected using the Tokoyasu method [58], placed on a nickel formvar/carbon coated grid and floated in a dish of PBS ready for immunolabeling. For immunolabeling of the sections, the grids were placed section side down on drops of 0.1 M ammonium chloride to quench untreated aldehyde groups, then blocked for nonspecific binding on 1% fish skin gelatin in PBS. Grids were incubated on a primary antibody rabbit anti-GFP. The grids were then placed on protein A gold 10 nm (UtrechtUMC). All grids were rinsed in PBS between steps, lightly fixed using 1% glutaraldehyde, rinsed and transferred to a UA/methylcellulose drop, then collected and dried. Grids were viewed in FEI Tencai Biotwin TEM at 80 Kv. Images were taken using Morada CCD and iTEM (Olympus) software.

### CERT binding partner identification

Lysates from HEK293 transfected with a 3xFLAG-CERT construct for 18 h and infected with *C. trachomatis* for an additional 24 h were immunoprecipitated using anti-FLAG M2 agarose beads. The bound proteins were separated by SDS-PAGE and analyzed by Silver Nitrate staining. A ∼15 kDa band present in the transfected/infected samples but not in the transfected or infected only samples was analyzed by LC MS-MS at the Yale W.M. Keck laboratory.

### Co-Immunoprecipitation

5.10^5^ HEK293 cells plated in 6-well tissue culture dishes and transfected for 48 h were washed once with 1× PBS and lyzed for 30 min in 300 µl of lysis buffer (20 mM Tris pH 7.5, 150 mM NaCl, 2 mM EDTA, 1%Triton X-100, 1 mM PMSF and protease inhibitor cocktail (Roche)). The lysates were centrifuge at 13,000 rpm for 10 min. An aliquot of the clarified lysate was collected (Input). The clarified lysates were incubated for 2 h in the presence of 10 µl of anti-FLAG M2 agarose beads (Sigma). The beads were washed 3 times (20 mM Tris pH 7.5, 150 mM NaCl, 2 mM EDTA, 1%Triton X-100) and the bound proteins were eluted with 15 µl of elution buffer (20 mM Tris pH 7.5, 150 mM NaCl, 2 mM EDTA, 100 µg/ml 3XFLAG peptide (Sigma)). 10 µl of the eluted fraction was collected (IP). All steps were conducted at 4°C.

### Protein purification

MBP and MBP-CERT PH were expressed in *E. coli* (BL2lDE3). The bacteria were resuspended in lysis buffer (20 mM Tris pH 7.5, 300 mM NaCl, 2 mM EDTA, 1 mM MgCl_2_, 1% Triton X-100, 1 mM DTT and 1 mM PMSF) and lysed by sonication. The clarified lysates were incubated for 2 h in the presence of amylose resin (NEB). The resin was washed 3 times with 20 mM Tris pH 7.5, 300 mM NaCl, 1 mM MgCl_2_. The bound proteins were eluted by incubation with 20 mM Tris pH 7.5, 100 mM NaCl, 1 mM MgCl_2_, 30 mM Gluthathione, dialysed over night in 20 mM Tris pH 7.5, 100 mM NaCl, 1 mM MgCl_2_ and stored in small aliquots at −20°C. GST and GST-IncD were prepared as described above and bound to glutathione sepharose beads (GE Healthcare). All steps were conducted at 4°C.

### *In vitro* binding assay

Equal amounts of freshly purified GST fusion proteins bound to glutathione beads were incubated with 0.2 µM of the indicated MBP fusion proteins in binding buffer (20 mM Tris pH 7.5, 150 mM NaCl, 1 mM MgCl_2_, 0.2% Triton X-100) for 2 h at 4°C, washed 3 times with 20 mM Tris pH 7.5, 300 mM NaCl, 1 mM MgCl_2_, 0.2% Triton X-100 and Laemli buffer, separated by SDS-PAGE and analyzed by immunoblot.

### Inclusion size quantification and computer-assisted image analysis

siRNA treated cells were infected with *C. trachomatis* for 36 hrs. The nuclei and the bacteria were labeled with the DNA dye Hoechst and the inclusions were counter-stained with a rabbit polyclonal antibody against *C. trachomatis* and a goat anti-rabbit AlexaFluor 594 antibody. The cells were subjected to automated fluorescence microscopy to capture images corresponding to the cell nuclei and the inclusion. Computer-assisted image analysis, using the analytical tools of the Metamorph software, was used to determine the number of nuclei and the surface area of each inclusion.

### Infectious progeny production

HeLa cells incubated with the indicated siRNA duplexes for 3 days were collected 48 h post infection, lysed with glass beads and dilutions of the lysate were used to infect fresh HeLa cells. The cells were fixed 24 h post infection and the number of inclusion forming units (IFUs) was determined after assessment of the number of infected cells by immonolabelling.

### BODIPYFL-C5-Ceramide labeling

Control and siRNA treated *C. trachomatis*-infected HeLa cells seeded onto glass coverslips were washed three times with cold Hank's Balanced Salt Solution (HBSS, Invitrogen) and incubated in DMEM containing 2.5 µM DFBSA/BODIPYFL-C5-Ceramide (Invitrogen) and 5 µg/ml Hoechst for 30 min at 4°C. The cells were then washed three times with cold HBSS and incubated in DMEM containing 0.34% DFBSA (Calbiochem) at 37°C in the presence of 5% CO_2_. Pictures of the live cells were acquired in the FITC and DAPI channel with a 10× objective every 15 min after the beginning of the chase.

### Accession numbers

The accession numbers for *C. trachomatis* (L2/434/Bu) proteins are IncD (CTL0370): CAP03810.1, IncE (CTL0371): CAP03811.1 and IncF (CTL0372): CAP03812.1.

The accession numbers for the mammalian genes are CERT (isoform 2): NM_031361.2, VAPA (isoform 2): NM_194434.2, VAPB: NM_004738.4, ARF1: NM_001024227, PI4KIIIß: NM_002651.1, OCRL1: NM_000276.3 and PI4KIIα: NM_018425.2.

## Supporting Information

Figure S1 **CERT localizes to the Golgi in uninfected cells but not in** ***C. trachomatis*** **infected cells.** (A–C) HeLa cells left uninfected (Uninfected) or infected with *C. trachomatis* for 24 h (*C. trachomatis*) were labeled with antibodies against CERT (CERT, green) (A–C) and the Golgi makers GM130 (GM130, red) (A), p115 (p115, red) (B) or TGN46 (TGN46, red) (C). The host cell nuclei and the bacterial DNA were labeled with the DNA dye Hoechst (DNA, blue). The merge images are shown in the right panels. Scale Bar, 10 µm.
(TIF)

Figure S2 **Inclusion localization of full length CERT or CERT depleted of its PH domain in control or VAPA/B-depleted cells.** Extended focus images, created from a stack of serial z-sections from HeLa cells depleted or not from VAPA&B (VAPA&B siRNA, bottom panels and CTRL siRNA, top and middle panels, respectively) expressing CERT-GFP fusion proteins (green), containing either full-length CERT (FL-CERT, top panels) or CERT deleted of its PH domain (CERT ΔPH, middle and bottom panels) and infected with *C. trachomatis* for 24 h. The cells were labeled with the inclusion membrane protein IncA (IncA, red). The merge images are shown on the right. Scale Bar, 10 µm.
(TIF)

Figure S3 ***C. trachomatis*** **infection of HEK293 cells.** HEK293 cells infected with *C. trachomatis* for 24 h were fixed and stained with anti-IncA antibodies (A) or anti-CERT antibodies (B). The DNA dye Hoechst labeled the host cell nuclei and the bacterial DNA (DNA, red). The merge images are shown on the right. Scale Bar, 50 µm. The replication of *Chlamydia* in HEK293 is comparable to replication in HeLa cells. Large inclusions are formed and both IncA and CERT localize to the inclusion membrane.
(TIF)

Figure S4 **Cellular localization of 3xFLAG-IncD, 3xFLAG -IncE and 3xFLAG –IncF.** HeLa cells expressing 3xFLAG-IncD (IncD, green), 3xFLAG-IncE (IncE, green) or 3xFLAG-IncF (IncF, green) were labeled with antibodies against the FLAG peptide (3xFLAG-Inc, green) and the ER protein Calnexin (Calnexin, red). The DNA dye Hoechst labeled the host cell nuclei (DNA, blue). The merge images are shown on the right. Scale Bar, 10 µm.
(TIF)

Figure S5 **IncD immuno-labeling of infected cells after fixation and permeabilization using 4%PFA and saponin, respectively.** HeLa cells infected with *C. trachomatis* for 24 h were fixed and permeabilized using 4% PFA and Saponin, respectively, and labeled with antibodies against the inclusion membrane protein IncD (IncD, green) and the inclusion membrane protein IncA (IncA, red). The host cell nuclei and the bacterial DNA were labeled with the DNA dye Hoechst (DNA, blue). The merge image is shown on the right. The asterisk indicates an IncD/IncA positive inclusion. The arrowheads indicate IncD negative, IncA positive inclusions. Scale Bar, 20 µm.
(TIF)

Figure S6 **Efficacy of CERT and VAPA and VAPB knock-down.** HeLa cells were transfected with control siRNA (GFPsi) or two different pools of CERT siRNA (CERTsi1 and CERTsi2) or a pool of siRNA against VAPA and VAPB (VAPA&Bsi) for 3 days. The silencing efficiency was evaluated at the transcript level by quantitative PCR (A) or at the protein level by western blot (B).
(TIF)

Figure S7 **CERT is no longer detected onto** ***C. trachomatis*** **inclusion in CERT-depleted cells.** HeLa cells transfected with control siRNA (GFPsi) or CERT siRNA (CERTsi1) or a pool of siRNA against VAPA and VAPB (VAPA&Bsi) for 3 days and infected with *C. trachomatis* for 24 h were labeled with antibodies against CERT (CERT, green). The host cell nuclei and the bacterial DNA were labeled with the DNA dye Hoechst (DNA, blue). The merge images are shown on the right. Scale Bar, 10 µm.
(TIF)

Figure S8 **CERT-GFP localization in** ***C. trachomatis*** **or** ***C. caviae*** **infected cells.** HeLa 229 cells expressing the CERT-GFP fusion protein (green) and infected with *C. trachomatis* (Top panels) or *C. caviae* (Bottom panels) for 24 h, were labeled with antibodies against the inclusion membrane protein IncA (IncA, red). The DNA dye Hoechst labeled the host cell nuclei and the bacterial DNA (DNA, blue). The merge images are shown on the right. Scale Bar, 10 µm.
(TIF)

Figure S9 **Golgi morphology of control, CERT- or VAPA/B-depleted cells.** HeLa cells, transfected with control siRNA (CTRL) or a pool of CERT siRNA (CERTsi) or a pool of siRNA against VAPA and VAPB (VAPA&Bsi) for 3 days, were labeled with antibodies against the Golgi maker GM130 (GM130, green). The host cell nuclei were labeled with the DNA dye Hoechst (DNA, blue). The merge images are shown in the right panels. Scale Bar, 10 µm.
(TIF)

Figure S10 **BODIPYFL-C5-Ceramide labeling of control cells infected with** ***C. trachomatis*** **.** HeLa cells transfected with control siRNA for 3 days and infected with *C. trachomatis* for 28 h were labeled with BODIPYFL-C5-Ceramide and Hoechst. The ceramide was chased for the indicated time and pictures were acquired in the DAPI (DNA, red) and FITC (BODIPYFL-C5-Ceramide, green) channels. The merge images are shown in the bottom panels. Asterisks indicate *C. trachomatis* inclusions. Scale Bar, 10 µm.
(TIF)

Figure S11 **BODIPYFL-C5-Ceramide labeling of CERT-depleted cells infected with** ***C. trachomatis*** **.** HeLa cells transfected with CERT siRNA for 3 days and infected with *C. trachomatis* for 28 h were labeled with BODIPYFL-C5-Ceramide and Hoechst. The ceramide was chased for the indicated time and pictures were acquired in the DAPI (DNA, red) and FITC (BODIPYFL-C5-Ceramide, green) channels. The merge images are shown in the bottom panels. Asterisks indicate *C. trachomatis* inclusions. Scale Bar, 10 µm.
(TIF)

Figure S12 **BODIPYFL-C5-Ceramide labeling of VAPA&B- depleted cells infected with** ***C. trachomatis*** **.** HeLa cells transfected with VAPA&B siRNA for 3 days and infected with *C. trachomatis* for 28 h were labeled with BODIPYFL-C5-Ceramide and Hoechst. The ceramide was chased for the indicated time and pictures were acquired in the DAPI (DNA, red) and FITC (BODIPYFL-C5-Ceramide, green) channels. The merge images are shown in the bottom panels. Asterisks indicate *C. trachomatis* inclusions. Scale Bar, 10 µm.
(TIF)

Figure S13 **Comparison of BODIPYFL-C5-Ceramide labeling of control cells, CERT-depleted cells and VAPA&B-depleted cells infected with** ***C. trachomatis*** **.** Images of representative inclusions shown in Supplementary Figures S10 (Control), S11 (CERT siRNA) and S12 (VAPA&B siRNA). DNA: Red, BODIPYFL-C5-Ceramide: Green. Asterisks indicate *C. trachomatis* inclusions. Scale Bar, 10 µm.
(TIF)

Table S1 **Sequence of the siRNA duplexes used in this study.**
(DOC)

Table S2 **Probes and Primers used for the quantitative PCR.** The probes and primers were designed according to Roche recommendation: http://www.roche-applied-science.com/sis/rtpcr/upl/index.jsp?id=uplct_030000.
(DOC)

Table S3 **Plasmids constructed for this study.** The following plasmids were constructed for this study. The table indicates the name of the inserts, the vectors and the restriction sites in which they were cloned into, and the sequences of the primers.
(DOC)
