# Supplementary figures and images for: The Lipid Transfer Protein CERT Interacts with the Chlamydia Inclusion Protein IncD and Participates to ER-Chlamydia Inclusion Membrane Contact Sites
Source: PLoS Pathog. 2011 Jun 23;7(6):e1002092. doi: 10.1371/journal.ppat.1002092 (PMC3121800; doi:10.1371/journal.ppat.1002092)

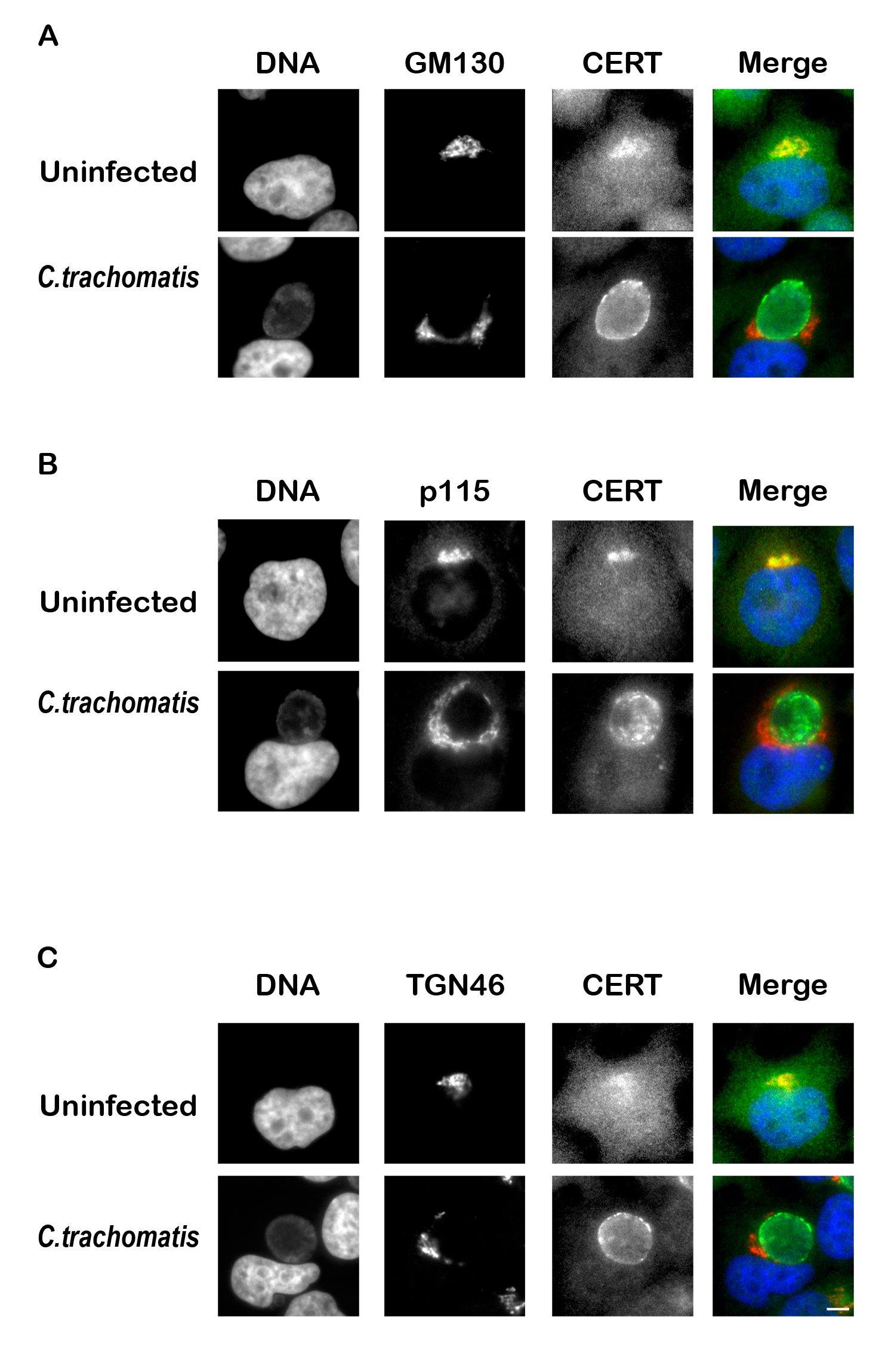

Supplement: Figure S1 — CERT localizes to the Golgi in uninfected cells but not in C. trachomatis infected cells. (A–C) HeLa cells left uninfected (Uninfected) or infected with C. trachomatis for 24 h (C. trachomatis) were labeled with antibodies against CERT (CERT, green) (A–C) and the Golgi makers GM130 (GM130, red) (A), p115 (p115, red) (B) or TGN46 (TGN46, red) (C). The host cell nuclei and the bacterial DNA were labeled with the DNA dye Hoechst (DNA, blue). The merge images are shown in the right panels. Scale Bar, 10 µm. (TIF) [file ppat.1002092.s001.tif]

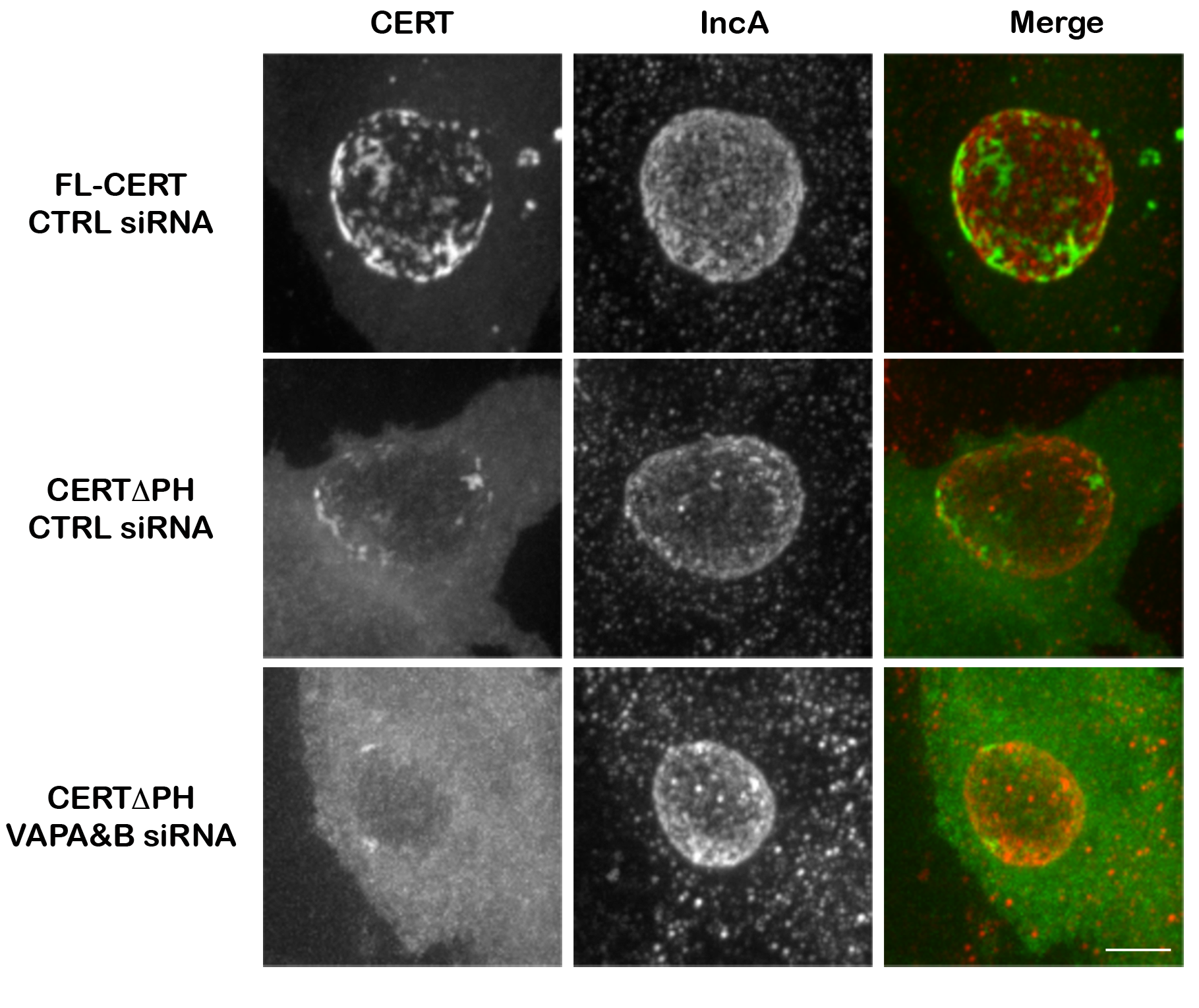

Supplement: Figure S2 — Inclusion localization of full length CERT or CERT depleted of its PH domain in control or VAPA/B-depleted cells. Extended focus images, created from a stack of serial z-sections from HeLa cells depleted or not from VAPA&B (VAPA&B siRNA, bottom panels and CTRL siRNA, top and middle panels, respectively) expressing CERT-GFP fusion proteins (green), containing either full-length CERT (FL-CERT, top panels) or CERT deleted of its PH domain (CERT ΔPH, middle and bottom panels) and infected with C. trachomatis for 24 h. The cells were labeled with the inclusion membrane protein IncA (IncA, red). The merge images are shown on the right. Scale Bar, 10 µm. (TIF) [file ppat.1002092.s002.tif]

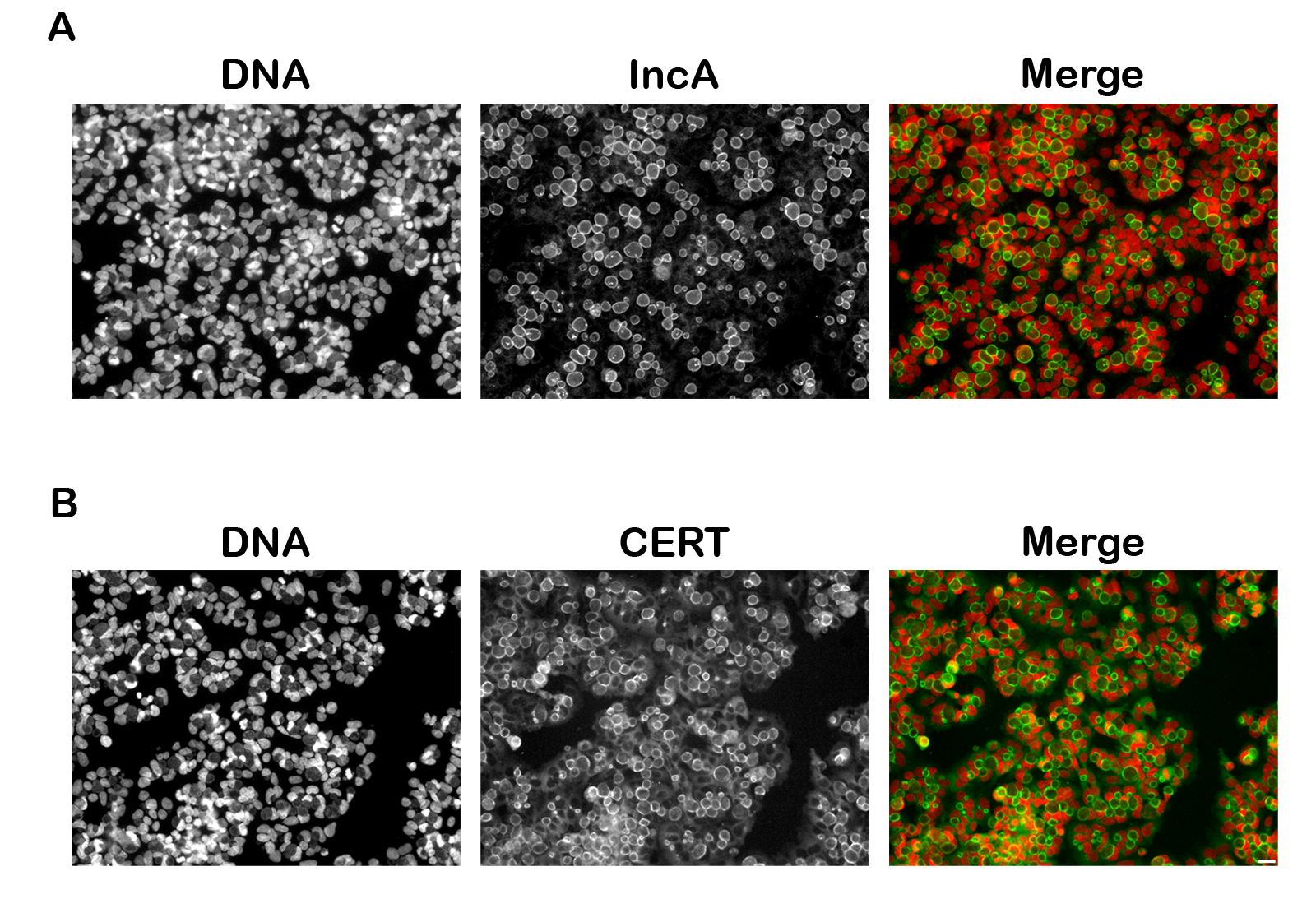

Supplement: Figure S3 — C. trachomatis infection of HEK293 cells. HEK293 cells infected with C. trachomatis for 24 h were fixed and stained with anti-IncA antibodies (A) or anti-CERT antibodies (B). The DNA dye Hoechst labeled the host cell nuclei and the bacterial DNA (DNA, red). The merge images are shown on the right. Scale Bar, 50 µm. The replication of Chlamydia in HEK293 is comparable to replication in HeLa cells. Large inclusions are formed and both IncA and CERT localize to the inclusion membrane. (TIF) [file ppat.1002092.s003.tif]

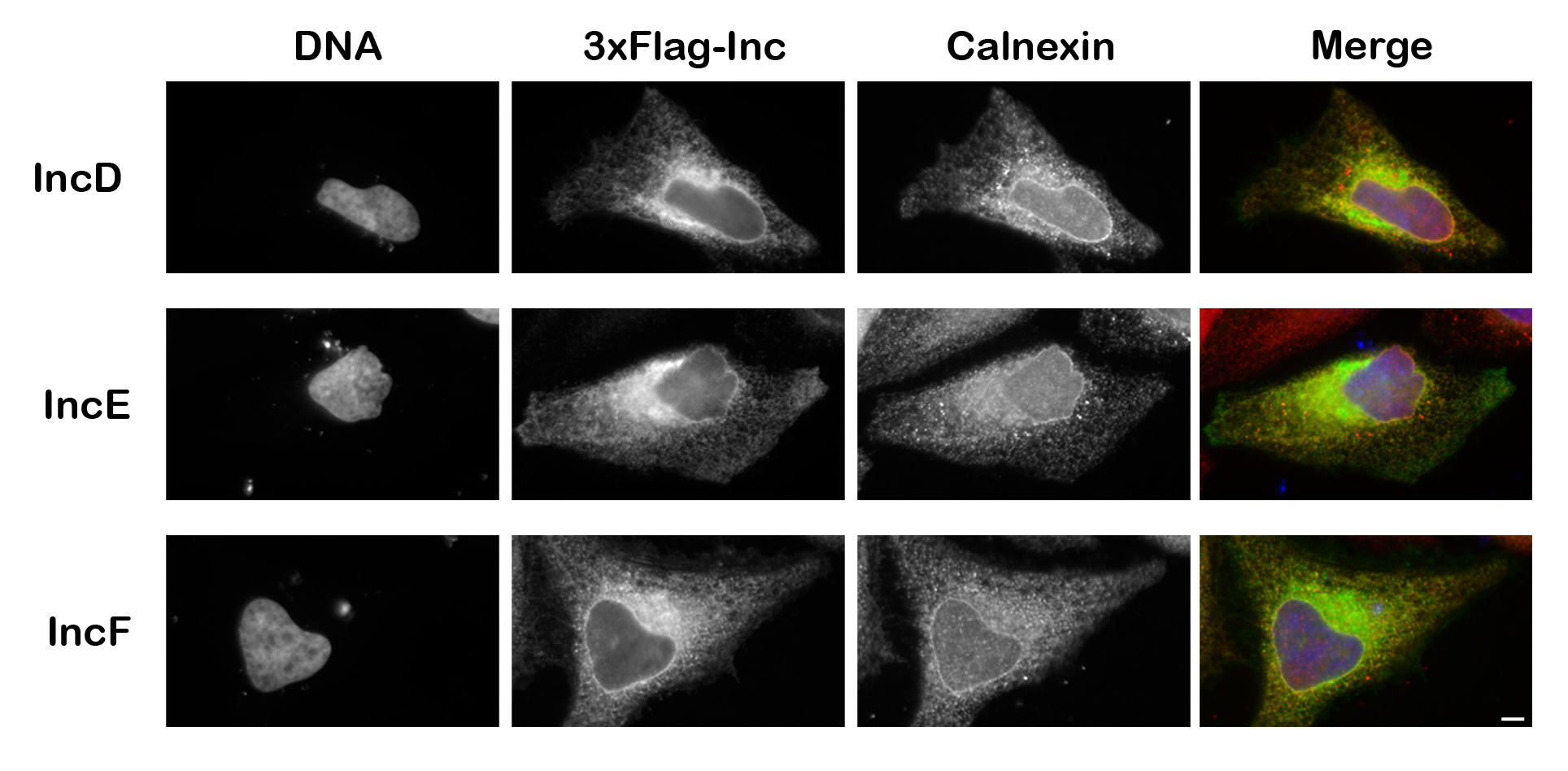

Supplement: Figure S4 — Cellular localization of 3xFLAG-IncD, 3xFLAG -IncE and 3xFLAG –IncF. HeLa cells expressing 3xFLAG-IncD (IncD, green), 3xFLAG-IncE (IncE, green) or 3xFLAG-IncF (IncF, green) were labeled with antibodies against the FLAG peptide (3xFLAG-Inc, green) and the ER protein Calnexin (Calnexin, red). The DNA dye Hoechst labeled the host cell nuclei (DNA, blue). The merge images are shown on the right. Scale Bar, 10 µm. (TIF) [file ppat.1002092.s004.tif]

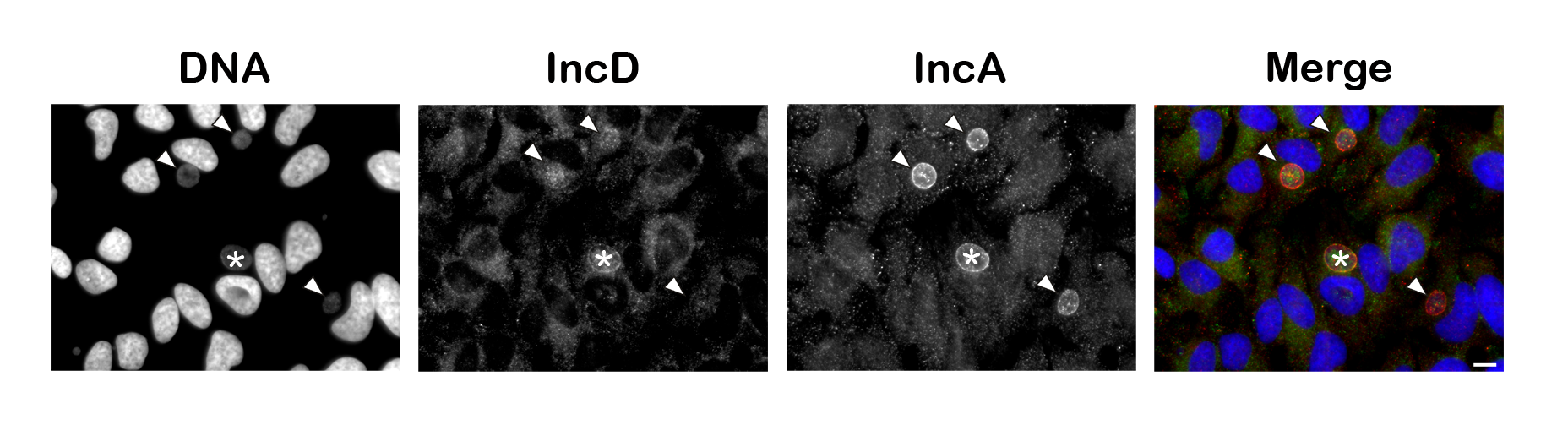

Supplement: Figure S5 — IncD immuno-labeling of infected cells after fixation and permeabilization using 4%PFA and saponin, respectively. HeLa cells infected with C. trachomatis for 24 h were fixed and permeabilized using 4% PFA and Saponin, respectively, and labeled with antibodies against the inclusion membrane protein IncD (IncD, green) and the inclusion membrane protein IncA (IncA, red). The host cell nuclei and the bacterial DNA were labeled with the DNA dye Hoechst (DNA, blue). The merge image is shown on the right. The asterisk indicates an IncD/IncA positive inclusion. The arrowheads indicate IncD negative, IncA positive inclusions. Scale Bar, 20 µm. (TIF) [file ppat.1002092.s005.tif]

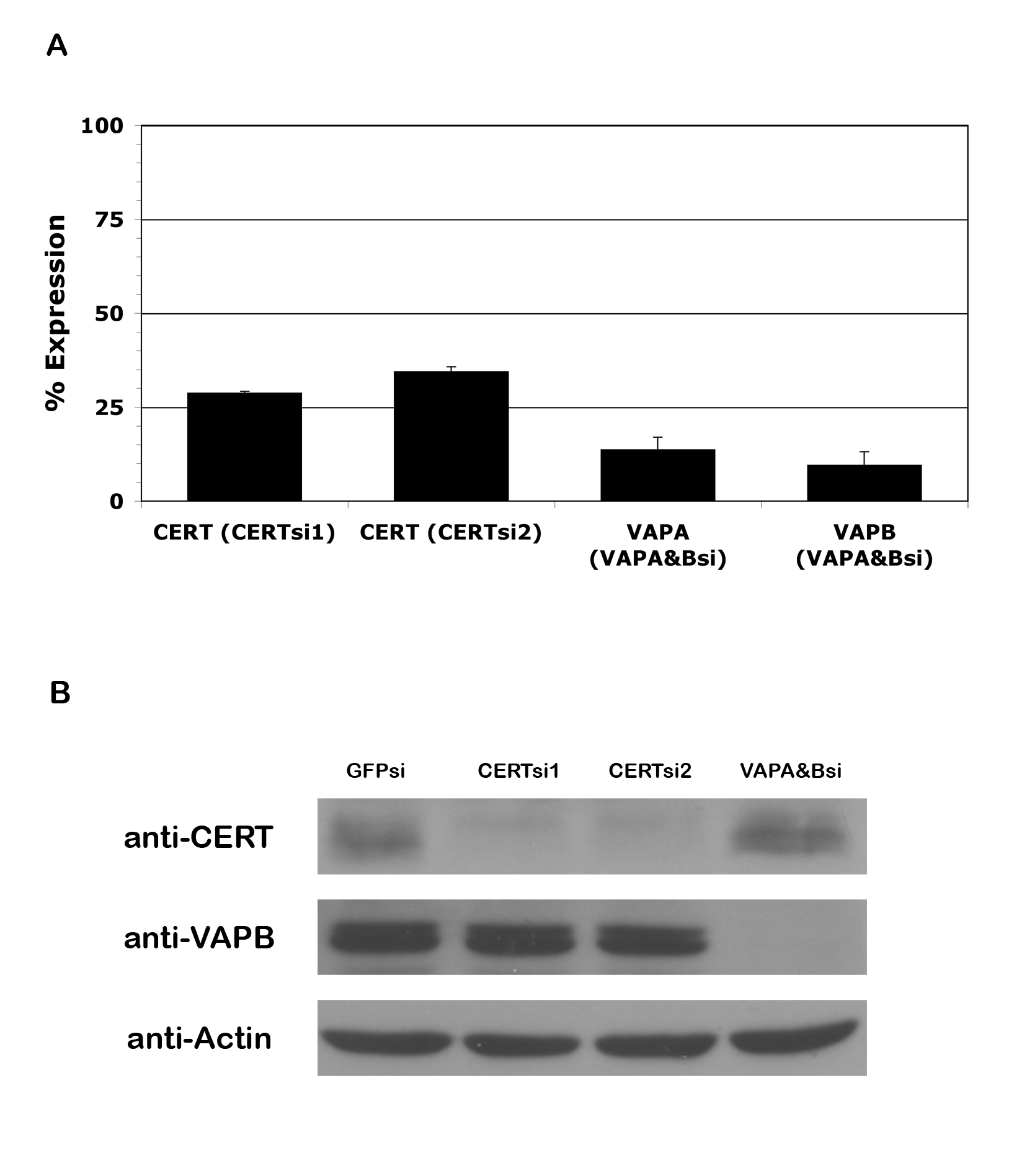

Supplement: Figure S6 — Efficacy of CERT and VAPA and VAPB knock-down. HeLa cells were transfected with control siRNA (GFPsi) or two different pools of CERT siRNA (CERTsi1 and CERTsi2) or a pool of siRNA against VAPA and VAPB (VAPA&Bsi) for 3 days. The silencing efficiency was evaluated at the transcript level by quantitative PCR (A) or at the protein level by western blot (B). (TIF) [file ppat.1002092.s006.tif]

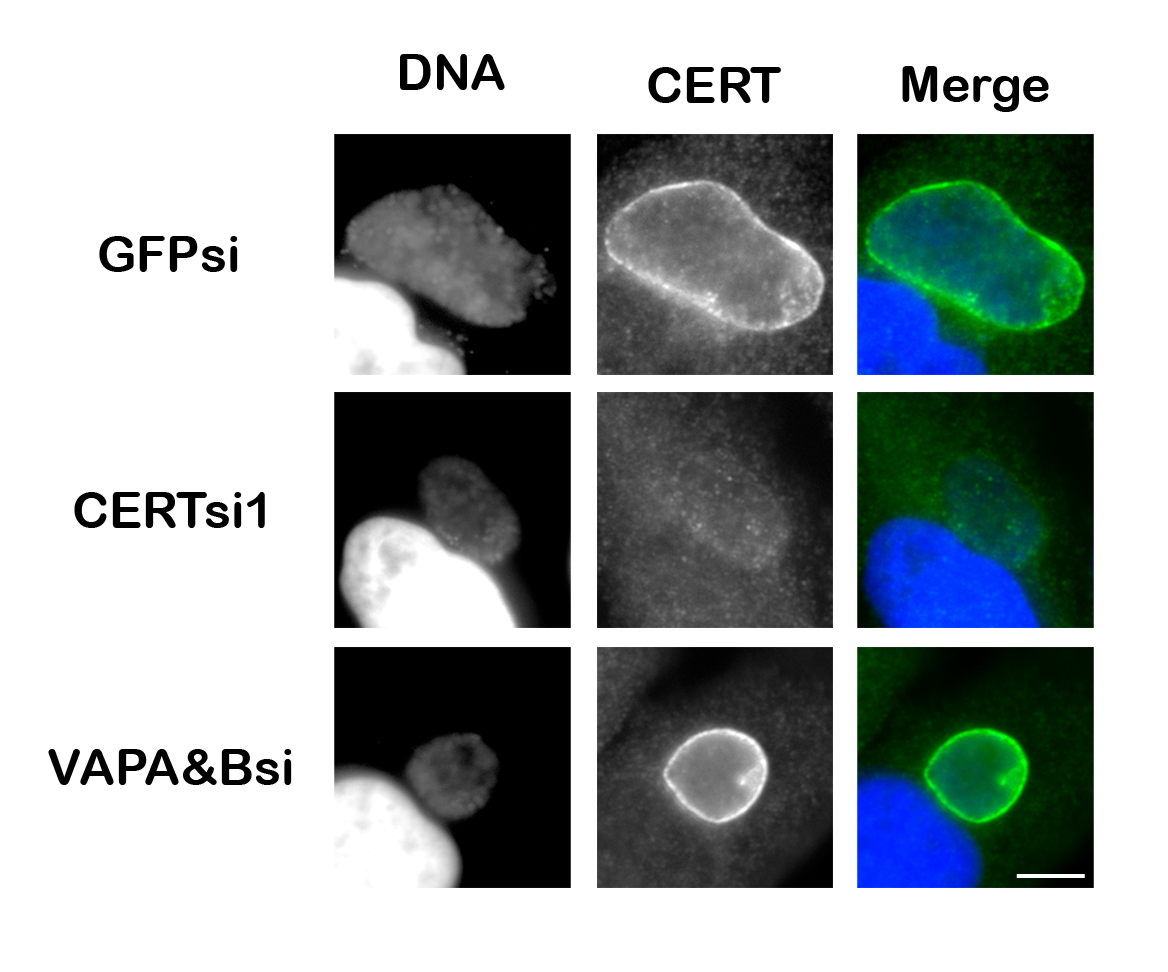

Supplement: Figure S7 — CERT is no longer detected onto C. trachomatis inclusion in CERT-depleted cells. HeLa cells transfected with control siRNA (GFPsi) or CERT siRNA (CERTsi1) or a pool of siRNA against VAPA and VAPB (VAPA&Bsi) for 3 days and infected with C. trachomatis for 24 h were labeled with antibodies against CERT (CERT, green). The host cell nuclei and the bacterial DNA were labeled with the DNA dye Hoechst (DNA, blue). The merge images are shown on the right. Scale Bar, 10 µm. (TIF) [file ppat.1002092.s007.tif]

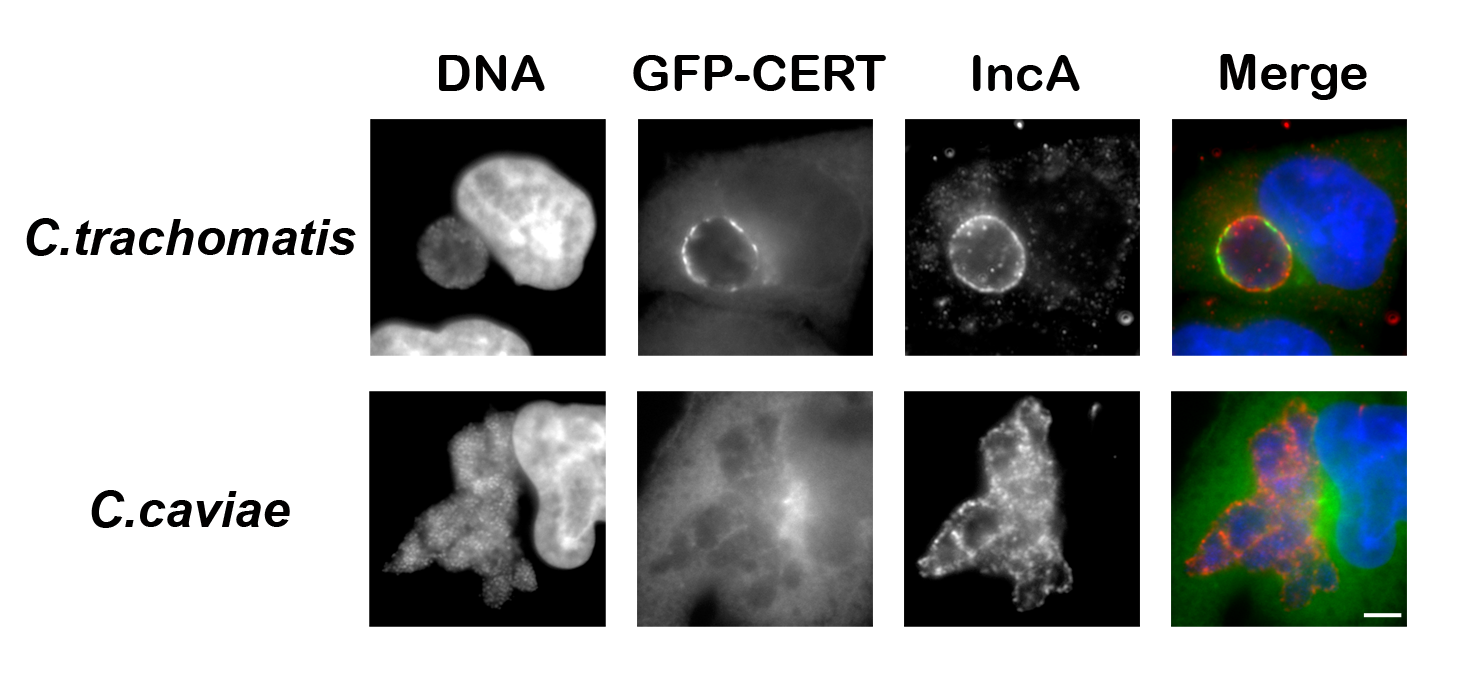

Supplement: Figure S8 — CERT-GFP localization in C. trachomatis or C. caviae infected cells. HeLa 229 cells expressing the CERT-GFP fusion protein (green) and infected with C. trachomatis (Top panels) or C. caviae (Bottom panels) for 24 h, were labeled with antibodies against the inclusion membrane protein IncA (IncA, red). The DNA dye Hoechst labeled the host cell nuclei and the bacterial DNA (DNA, blue). The merge images are shown on the right. Scale Bar, 10 µm. (TIF) [file ppat.1002092.s008.tif]

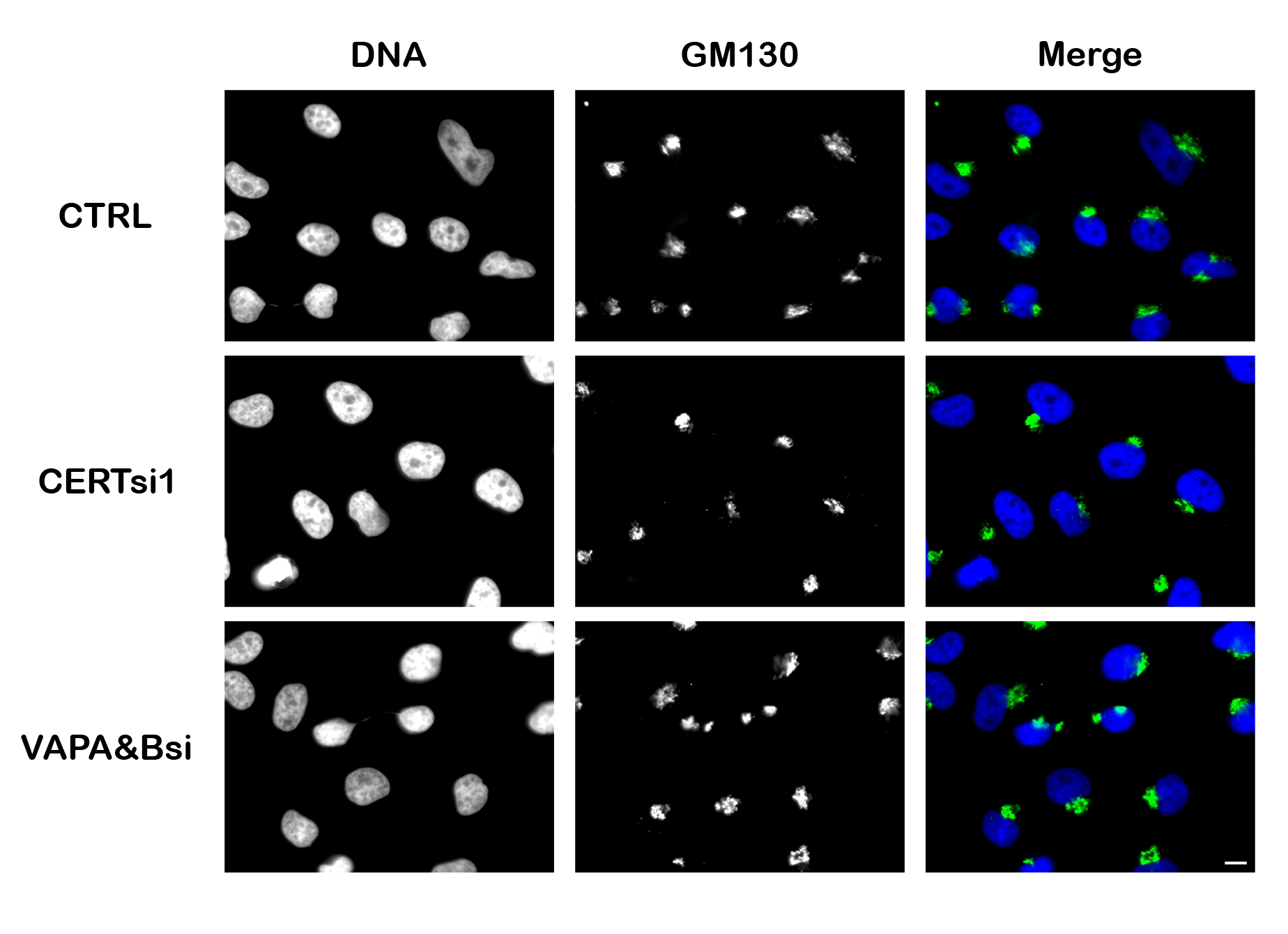

Supplement: Figure S9 — Golgi morphology of control, CERT- or VAPA/B-depleted cells. HeLa cells, transfected with control siRNA (CTRL) or a pool of CERT siRNA (CERTsi) or a pool of siRNA against VAPA and VAPB (VAPA&Bsi) for 3 days, were labeled with antibodies against the Golgi maker GM130 (GM130, green). The host cell nuclei were labeled with the DNA dye Hoechst (DNA, blue). The merge images are shown in the right panels. Scale Bar, 10 µm. (TIF) [file ppat.1002092.s009.tif]

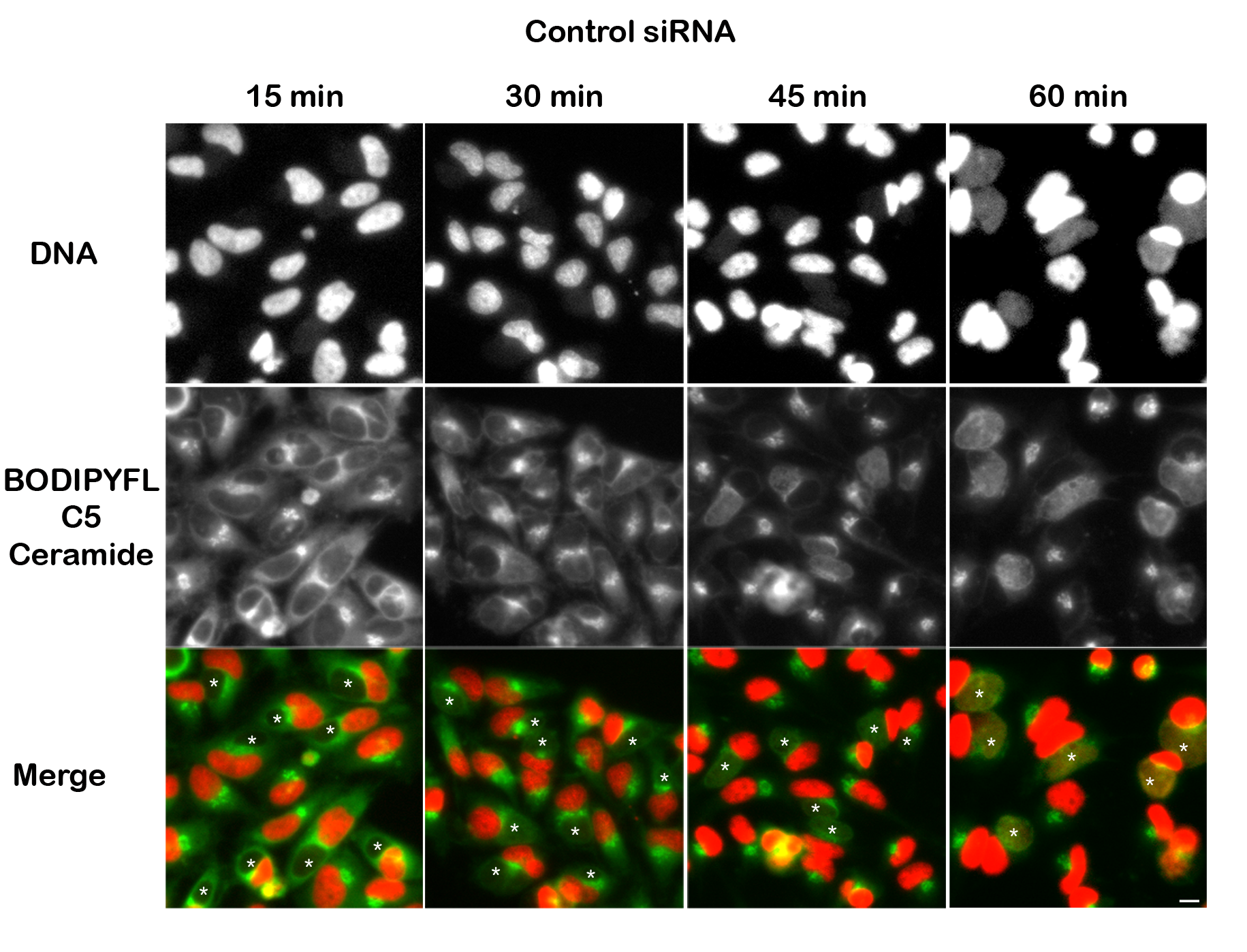

Supplement: Figure S10 — BODIPYFL-C5-Ceramide labeling of control cells infected with C. trachomatis . HeLa cells transfected with control siRNA for 3 days and infected with C. trachomatis for 28 h were labeled with BODIPYFL-C5-Ceramide and Hoechst. The ceramide was chased for the indicated time and pictures were acquired in the DAPI (DNA, red) and FITC (BODIPYFL-C5-Ceramide, green) channels. The merge images are shown in the bottom panels. Asterisks indicate C. trachomatis inclusions. Scale Bar, 10 µm. (TIF) [file ppat.1002092.s010.tif]

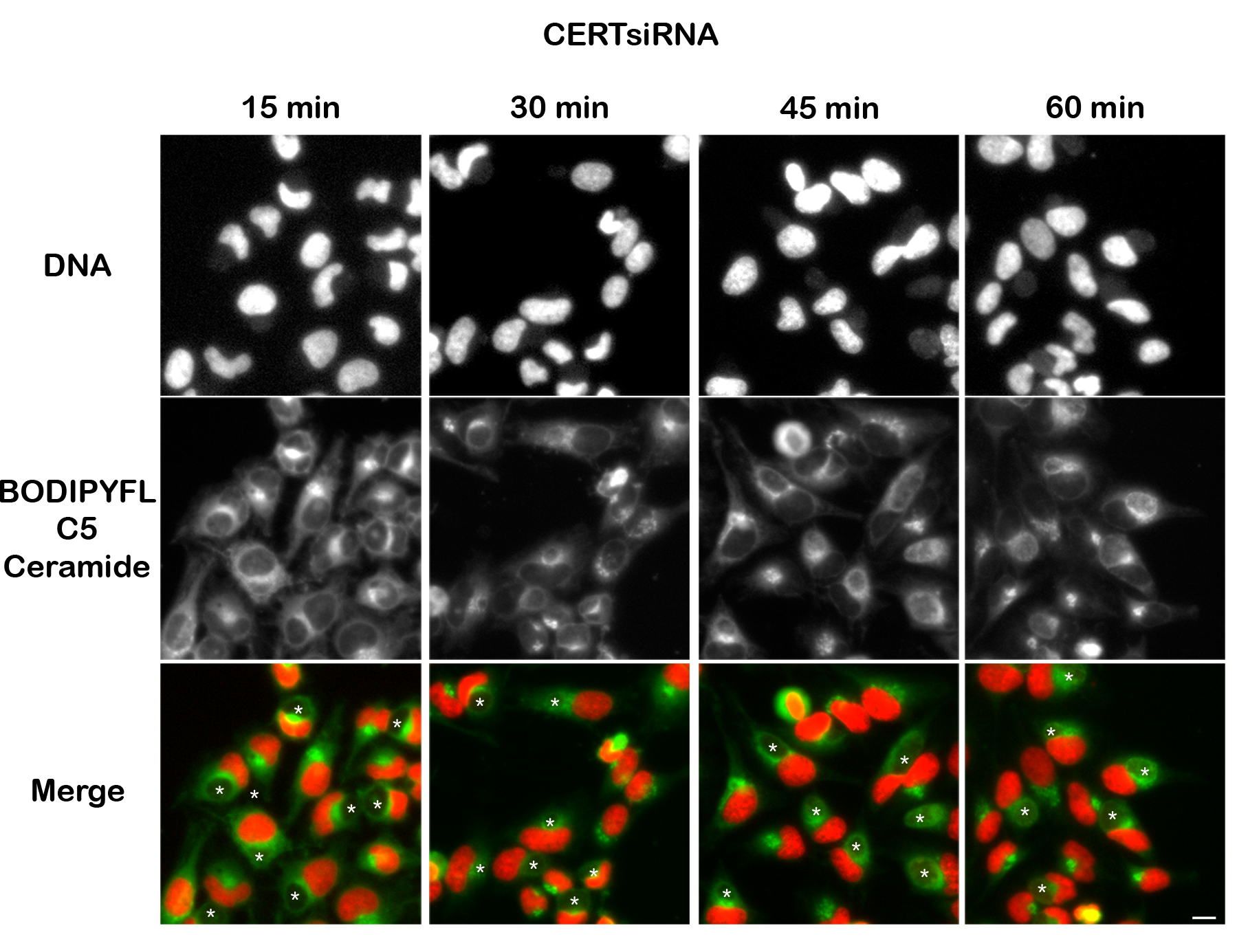

Supplement: Figure S11 — BODIPYFL-C5-Ceramide labeling of CERT-depleted cells infected with C. trachomatis . HeLa cells transfected with CERT siRNA for 3 days and infected with C. trachomatis for 28 h were labeled with BODIPYFL-C5-Ceramide and Hoechst. The ceramide was chased for the indicated time and pictures were acquired in the DAPI (DNA, red) and FITC (BODIPYFL-C5-Ceramide, green) channels. The merge images are shown in the bottom panels. Asterisks indicate C. trachomatis inclusions. Scale Bar, 10 µm. (TIF) [file ppat.1002092.s011.tif]

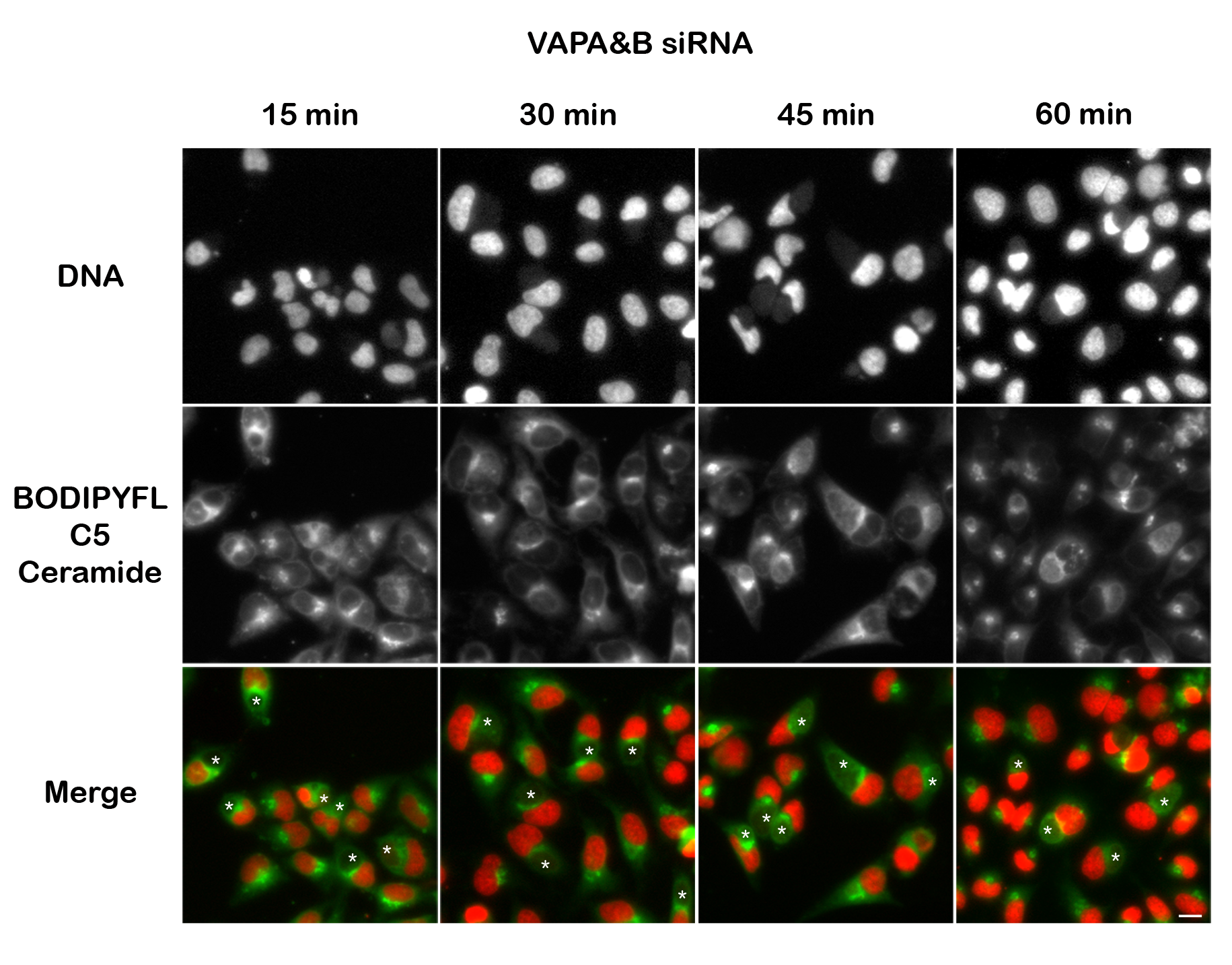

Supplement: Figure S12 — BODIPYFL-C5-Ceramide labeling of VAPA&B- depleted cells infected with C. trachomatis . HeLa cells transfected with VAPA&B siRNA for 3 days and infected with C. trachomatis for 28 h were labeled with BODIPYFL-C5-Ceramide and Hoechst. The ceramide was chased for the indicated time and pictures were acquired in the DAPI (DNA, red) and FITC (BODIPYFL-C5-Ceramide, green) channels. The merge images are shown in the bottom panels. Asterisks indicate C. trachomatis inclusions. Scale Bar, 10 µm. (TIF) [file ppat.1002092.s012.tif]

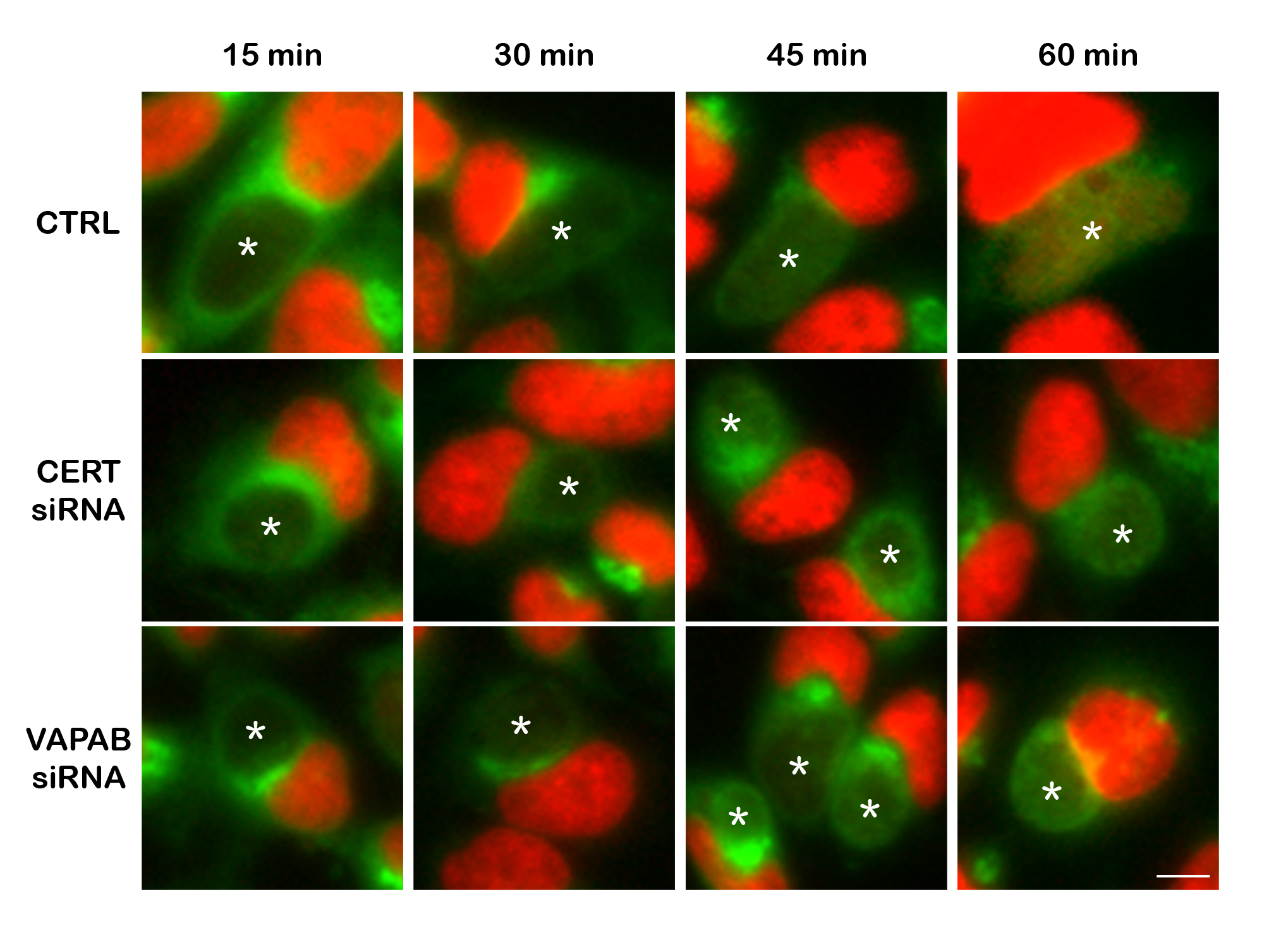

Supplement: Figure S13 — Comparison of BODIPYFL-C5-Ceramide labeling of control cells, CERT-depleted cells and VAPA&B-depleted cells infected with C. trachomatis . Images of representative inclusions shown in Supplementary Figures S10 (Control), S11 (CERT siRNA) and S12 (VAPA&B siRNA). DNA: Red, BODIPYFL-C5-Ceramide: Green. Asterisks indicate C. trachomatis inclusions. Scale Bar, 10 µm. (TIF) [file ppat.1002092.s013.tif]
