# Supplementary material for: The Lipid Transfer Protein CERT Interacts with the Chlamydia Inclusion Protein IncD and Participates to ER-Chlamydia Inclusion Membrane Contact Sites
Source: PLoS Pathog. 2011 Jun 23;7(6):e1002092. doi: 10.1371/journal.ppat.1002092 (PMC3121800; doi:10.1371/journal.ppat.1002092)
Supplement: Table S1 — Sequence of the siRNA duplexes used in this study. (DOC) [file ppat.1002092.s014.doc]

| **Targeted gene** | **Sequence** |
| --- | --- |
| CERT (si1) | GAAGAUGACUUUCCUACAA, GAAGUUGGCUGAAAUGGAA, GCGAGAGUAUCCUAAAUUU, UCAAAGGGAUAAAGUGGUA |
| CERT (si2) | GAACAGAGGAAGCAUAUAA, GAUGGUGACUUCUUGCAUA, GUGGAAACAUUAGCUGAUA, GAACAGCACAAGACUGAAU |
| VAPA | CCACAGACCUCAAAUUCAA, GGCAAAACCUGAUGAAUUA, CCUGAGAGAUGAAGGUUUA, CAAGGAAACUAAUGGAAGA |
| VAPB | GUAAGAGGCUGCAAGGUGA, CCACGUAGGUACUGUGUGA, UGUUACAGCCUUUCGAUUA, GUAAUUAUUGGGAAGAUUG |
