# Supplementary material for: The Lipid Transfer Protein CERT Interacts with the Chlamydia Inclusion Protein IncD and Participates to ER-Chlamydia Inclusion Membrane Contact Sites
Source: PLoS Pathog. 2011 Jun 23;7(6):e1002092. doi: 10.1371/journal.ppat.1002092 (PMC3121800; doi:10.1371/journal.ppat.1002092)
Supplement: Table S2 — Probes and Primers used for the quantitative PCR. The probes and primers were designed according to Roche recommendation: http://www.roche-applied-science.com/sis/rtpcr/upl/index.jsp?id=uplct_030000. (DOC) [file ppat.1002092.s015.doc]

| **Target** | **Probe #** | **Forward Primer** | **Reverse Primer** |
| --- | --- | --- | --- |
| GAPDH | 60 | agccacatcgctcagacac | gcccaatacgaccaaatcc |
| CERT | 50 | cgacatggctcaatggtgt | tcacgtaaactgtggcctttc |
| VAPA | 22 | ccctttgactatgatccgaatg | ggttttgcctctttccacac |
| VAPB | 38 | tttgctccaactgacacttca | ccataaggtcttccggttttg |
