# Supplementary material for: The Lipid Transfer Protein CERT Interacts with the Chlamydia Inclusion Protein IncD and Participates to ER-Chlamydia Inclusion Membrane Contact Sites
Source: PLoS Pathog. 2011 Jun 23;7(6):e1002092. doi: 10.1371/journal.ppat.1002092 (PMC3121800; doi:10.1371/journal.ppat.1002092)
Supplement: Table S3 — Plasmids constructed for this study. The following plasmids were constructed for this study. The table indicates the name of the inserts, the vectors and the restriction sites in which they were cloned into, and the sequences of the primers. (DOC) [file ppat.1002092.s016.doc]

| **Plasmid** | **Insert** | **Cloned into** | **Forward primer**  **Reverse Primer** |
| --- | --- | --- | --- |
| **CERT-GFP** | **CERT** | pEGFPN1  EcoRI/AgeI | GAAGAATTCatgtcggataatcagagctgg  ACCACCGGTGGgaacaaaataggctttcctgc |
| **3xFLAG-CERT** | **CERT** | p3XFLAG  EcoRI/XhoI | GAAGAATTCTatgtcggataatcagagctgg  CTCCTCGAGctagaacaaaataggctttcctgc |
| **CERT PH-GFP** | **CERT PH** | pEGFPN1  EcoRI/AgeI | GAAGAATTCATGgtggagcgctgcggggtcc  ACCACCGGTGGcttgtgctgttcaatggc |
| **MBP-CERT PH** | **CERT PH** | pMal  NdeI/EcoRI | CATCATATGgtggagcgctgcggggtcc  GAAGAATTCTTActtgtgctgttcaatggc |
| **CERT ∆PH-GFP** | **CERT ∆PH** | pEGFPN1  EcoRI/AgeI | GAAGAATTCATGgaatccagcttgcgtcgacatggc  ACCACCGGTGGgaacaaaataggctttcctgc |
| **VAPA-GFP** | **VAPA** | pEGFPN1  HindIII/AgeI | AAGAAGCTTatggcgtccgcctcaggggccatggcg  ACCACCGGTGGcaagatgaatttccctagaaag |
| **VAPB-GFP** | **VAPB** | pEGFPN1  EcoRI/AgeI | GAAGAATTCatggcgaaggtggagcaggtcc  ACCACCGGTGGcaaggcaatcttcccaataattac |
| **3xFLAG-IncD** | **IncD** | p3XFLAG  BamHI/XhoI | GGAGGATCCatgacgaaggtttatgcgc  CTCCTCGAGttagctcgccccttttttactc |
| **GST-IncD** | **IncD** | pGEXKG  BamHI/XhoI | GGAGGATCCatgacgaaggtttatgcgc  CTCCTCGAGttagctcgccccttttttactc |
| **3xFLAG-IncE** | **IncE** | p3XFLAG  EcoRI/XhoI | GAAGAATTCTatggaatgcgttaaacagttatg  CTCCTCGAGttattgagttactaaaatcac |
| **3xFLAG-IncF** | **IncF** | p3XFLAG  EcoRI/XhoI | GAAGAATTCTatgggagacgtgatgatacag  CTCCTCGAGctagcacttatttgtagaagc |
